# Registered Report: Testing Ideological Asymmetries in Measurement Invariance

**DOI:** 10.1177/1073191120983891

**Published:** 2021-01-29

**Authors:** Mark J. Brandt, Jia He, Michael Bender

**Affiliations:** 1 Michigan State University, East Lansing, MI, USA; 2 Tilburg University, Tilburg, Netherlands

**Keywords:** ideology, invariance, personality, ideological asymmetry, political psychology, measurement

## Abstract

People with different ideological identities differ in their values, personality, affect, and psychological motivations. These differences are observed on measures of practical and clinical importance and these differences are the central node tying together theories about the psychology of political ideology; however, they rest on a critical untested assumption: The measures are invariant across ideological groups. Here, we test this assumption across 28 constructs in data from the United States and the Netherlands. Measures are not invariant across ideological divisions. At the same time, estimates of ideological similarities and differences are largely similar before and after correcting for measurement noninvariance. This may give us increased confidence in the results from this research area, while simultaneously highlighting that some instance of noninvariance did change conclusions and that individual items are not always comparable across political groups.

People with different ideological identities and outlooks differ in the policies they support and the values they uphold (Graham et al., 2009; Miles & Vaisey, 2015). This is to be expected. Perhaps less expected is that people with different ideological identities would also differ in their personalities, as well as their needs for certainty and psychological security. Yet this is the case (for reviews, see Gerber et al., 2011; Jost, 2017). When comparing liberals (and leftists) and conservatives (and rightists), psychologists and political scientists find consistent evidence that liberals are less authoritarian (Altemeyer, 1998; Chistopher & Mull, 2006; Jost et al., 2008), less socially dominant (Christopher & Mull, 2006; Federico & Sidanius, 2002; Pratto et al., 1994; Pratto et al., 1998; Sidanius & Pratto, 1999), less racist (Blatz & Ross, 2009; Federico & Sidanius, 2002; Jost et al., 2008; Reyna et al., 2006; Tesler, 2012), less conscientious (Chirumbolo & Leone, 2010; Xu et al., 2013), and more open to experiences (Carney et al., 2008; Xu et al., 2013) compared with conservatives (for relevant meta-analyses, see Jost et al., 2003; Sibley & Duckitt, 2008; and Sibley et al., 2012). Although the results are not as consistent (Conway et al., 2016; Malka et al., 2014), conservatives also tend to be more motivated by fear and feelings of uncertainty compared with liberals (for meta-analyses, see Jost et al., 2003; Jost, Sterling, et al., 2017; and Jost, Stern, et al., 2017), tend to be less integrative thinkers (Tetlock, 1983; Tetlock et al., 1984), and tend to be happier and more satisfied with life (Napier & Jost, 2008; for a meta-analysis suggesting that such results are not robust, see Onraet et al., 2013). These ideological differences, which have been catalogued in hundreds of studies with hundreds of thousands of participants, are the essence of political psychology (Jost, 2017).

Ideological differences, from values and morality to basic personality traits, suggest that people are attracted to belief systems that resonate with their psychological needs and traits (Caprara & Zimbardo, 2004; Jost et al., 2009). This link has deep theoretical implications. It provides the stepping stones for describing how political beliefs are linked with biological responses and are (partially) genetically determined (Kandler et al., 2012; Ksiazkiewicz et al., 2016; Smith et al., 2011). It provides an individual-level explanation for ideological constraint (i.e., why some policies tend to correlate; Gerber et al., 2010). And it provides explanations for why people adopt belief systems that are contrary (or at least are not fully in line) with their material self-interest (Jost et al., 2003). Ideological differences in personality, motivations, and values are the central node tying together theories about the psychology of political ideology.

These ideological differences are not necessarily just theoretical. They have been catalogued using measures important in applied and clinical contexts (e.g., Big Five personality, life satisfaction, need for cognition, need for cognitive closure, negative affect, self-esteem). One implication of work on ideological differences is that ideological differences will also emerge in applied domains. Just as it is important to investigate gender, ethnic, and religious differences in applied domains, so too is it important to study political differences.

## The Hidden Assumption

All of the work on self-reported ideological differences relies on a hidden assumption: These studies assume that the measures mean the same thing to both liberals and conservatives. If they do not, that is, if psychometric properties of the measures are not invariant, it means that we cannot validly compare ideological groups. In such a case, any differences (or similarities) found could be attributable to differential responding to the measure itself, and cannot be linked to the target concept that was supposedly assessed (for a similar argument applied to personality and gender, see Ock et al., 2020; applied to personality and ethnicity, see Lui et al., 2020). Measurement invariance refers to comparable psychometric properties of measures administered to different discrete groups (or the same group repeatedly) or across levels of a continuous grouping variable. It requires empirical assessment for measurement invariance to be established and reestablished among groups under investigation (van de Vijver & Leung, 1997). Yet, in our reading, it appears to be largely ignored in the literature on ideological differences (see also Flake et al., 2017).

## The Necessity of Measurement Invariance

Measurement invariance for tests of ideological differences is a necessity. An analogy can be drawn to work comparing samples from different cultures. Such cross-cultural research prizes measurement invariance because it allows researchers to directly compare cultures on the measure of interest (e.g., happiness, values, or personality; Bieda et al., 2017; Davidov et al., 2008; Nye et al., 2008). However, in the face of measurement noninvariance, such comparisons are not possible because people from different cultures interpret particular words and phrases, scale items, or even the entire scale differently based on language differences or cultural assumptions. This occurs when constructs have different meanings, stimuli have different familiarity, and specific words are incomparable between groups (e.g., idioms; He & van de Vijver, 2012; van de Vijver & Leung, 1997). For example, happiness may not always be cross-culturally equivalent because happiness, as both a word and a broad construct, may have different connotations across cultures (e.g., maximization of positive affect vs. an equilibrium of positive and negative affect, Uchida et al., 2004). In short, target constructs, methods, and item content can be either equivalent or biased across different groups.

Similarly, liberals and conservatives may have different background assumptions about the meaning of the construct of interest, the response options, and items, as well as systematic differences in their use of the response scales. If such systematic differences exist in domains of personality, life satisfaction, self-esteem, and so on, it would complicate the assessment of these constructs in politically diverse samples. Indeed, we know that ideological differences lead to different interpretations of scientific evidence (Kahan, 2012; Washburn & Skitka, 2018) and behavioral nudges (Tannenbaum et al., 2017); it is only a small jump to expect that ideological differences will also lead to different interpretations of psychological measurements.

Although measurement invariance was not specifically assessed, one related example comes from work on actively open-minded thinking and religiosity (Stanovich & Toplak, 2019). Typically, scholars find very strong negative correlations between religiosity and actively open-minded thinking; however, it turns out that this is because religious people interpret belief revision items (e.g., “Beliefs should always be revised in response to new information or evidence.”) very differently compared with less religious people. When these items are fixed (e.g., by rewording them) or removed, the correlation between religiosity and actively open-minded thinking is substantially reduced.

Ignoring the issues of measurement invariance between ideological groups can have severe consequences. If the scale scores of a target construct are overestimated for one group of respondents and underestimated for another group, or the data on this measure also contain differences in other constructs (e.g., social desirability) unrelated to the target construct, observed score differences do not accurately reflect genuine differences in the target construct. Cross-group comparisons are then invalid: mean difference is equivocal, and structural relations with other constructs may be masked, exaggerated, or simply a fluke due to measurement noise. By overlooking measurement invariance, we do not know if the theoretically consequential differences between liberals and conservatives (highlighted above) are meaningful and we do not know if differences not previously identified emerge after accounting for measurement noninvariance. More practically, societies consist of people with both liberal and conservative leanings. Approximately 60% of the United States identifies as liberal or conservative (Saad, 2019). If our psychological assessments function differently for these groups, it means that our assessments in basic research and clinical assessments need to account for such measurement noninvariance to be effective in the population as a whole.

In the analyses that follow, we will consider how self-identified political ideology is related to the latent psychological constructs of interest and whether and how it relates to item parameters in the measurement model of each target construct. In short, we see if there are ideological differences in the intercepts and factor loadings of observed indicators of a construct holding constant participants’ position on the latent construct. Ideological differences in the observed indicators independent of the latent construct are indicators of differential item functioning (DIF) and indicate measurement noninvariance (e.g., Zumbo, 2007).

## The Current Study

We test measurement invariance between self-identified liberals and conservatives on a variety of psychological measures. The measures include personality traits (e.g., Big Five personality), motivations (e.g., need for cognition, need for cognitive closure), and indicators of well-being (e.g., positive affect, negative affect, self-esteem) as well as more politically proximal measures (e.g., racism, social dominance orientation, belief in a just world). By including a range of measures we can assess if DIF (i.e., measurement noninvariance) only emerges on a subset of measures or if it is found more broadly, which will give us insight into the reasons for the DIF. Moreover, if DIF arises on the diverse array of measures we include, it makes it more plausible that it will arise on a variety of measures we do not include (e.g., clinical indicators).

If the extremity of ideology differs between liberals and conservatives in the sample (e.g., more extreme liberals than extreme conservatives), differences attributed to ideology may be attributable to ideological extremity rather than ideological direction. This is consistent with some work that finds that some psychological constructs may differ more due to levels of ideological extremity than ideological direction (Frimer et al., 2019; Sterling et al., 2020; Tetlock, 1984; Toner et al., 2013). Therefore, we will also include ideological extremity as a comparison with the results from analyses on ideological direction. We will also compare the association between ideology and the target constructs both before and after accounting for DIF to assess the extent correcting for DIF alters conclusions about ideological differences (for a conceptually similar approach studying personality and gender, see Ock et al., 2020).

If we find measurement invariance (absence of DIF) among respondents’ political ideology and the extremity of their political stance, then our confidence in previously reported ideological differences and the theoretical implications of that research is solidified. However, there is good reason to suspect that we will not find measurement invariance because a lack of scalar invariance appears to be the norm across many different psychological measures (e.g., across cultural groups see Zercher et al., 2015). If measures are not invariant between liberals and conservatives, then previous research that has aimed to identify differences between liberals and conservatives on self-reported psychological constructs cannot be interpreted because the origin of the difference will be unclear. This includes *both* measures in which there are robust ideological differences *and* measures where ideological similarities are typically obtained. In either case, without empirical demonstration of measurement invariance, the comparisons are at best ambiguous and at worst misleading. Importantly, items flagged with DIF are themselves an indicator of psychological differences: It may be tempting to treat DIF as a nuisance (Fischer & Poortinga, 2018; Poortinga, 1989), but it may in fact point toward meaningful systematic variation across (ideological) groups and how they interpret the world. We will begin to describe such differences by exploring potential causes of any DIF we find. The Stage 1 version of this article and the preregistration can be found here: https://osf.io/vutbg. We report how we determined our sample size, all data exclusions, all manipulations, and all measures in the study.

## Method

We assess ideological measurement invariance in two large samples. The first is a large convenience sample from the United States which includes 24 scales and subscales measuring psychological constructs ranging from well-being (e.g., self-esteem) to political values (e.g., social dominance orientation). This sample is a convenience sample, but consists of a large and diverse set of respondents from a considerable range of age groups and regions in the United States. It also includes many different relevant measures and uses a sampling strategy similar to highly cited work on ideological differences (e.g., Graham et al., 2009; Jost et al., 2008). The second is a large representative sample from the Netherlands which includes 11 scales and subscales measuring psychological constructs ranging from well-being (e.g., life satisfaction) to psychological motivations (e.g., need for cognition). We choose to use samples from distinct political systems because some work suggests that the link between ideology and psychological characteristics can vary across countries (Malka et al., 2014; Sibley et al., 2012). This suggests that DIF may also vary across countries. Notably, due to the different sampling strategies and administration, it is not possible to directly compare the samples (e.g., in a multigroup analysis); however, the two samples allow us to test measurement invariance and account for DIF between ideological groups in two distinct political systems across a diverse range of measures, testing the generalizability and robustness of our findings. We also assess if the results are qualitatively similar in both samples given that some scales are included in both samples (e.g., need for cognition, self-esteem). This builds replication into our study design.

As of the writing of the Stage 1 proposal of this registered report, we only had access to an exploratory subsample of the data set from the United States. We used this data set to calculate expected sample size. For the Dutch data set, we had analyzed data from this data set before for other projects, including some of the measures we are including here. However, we had never conducted the analyses we proposed. The methodological and analysis plan from the Stage 1 proposal was followed without any deviations. One reviewer in the second stage of review suggested to add *r* (based on *t*-test statistics and degree of freedom) as the effect size measure for DIF. We incorporated this suggestion in the second round of revisions of the Stage 2 article.

### United States Data Set

The Attitudes 2.0 data set (Hussey et al., 2018) provides information on self-identified liberals and conservatives in the United States and their responses to a variety of self-reported measures that have been tested for ideological differences. This allows us to check measurement invariance among ideological groups on a large array of variables. This data set is from the Attitudes, Identities, and Individual Differences Study carried out from 2004 to 2007. Data were collected via the Project Implicit research website, with the goal to examine the validity of constructs ranging from personality, values, and attitudes in a large-scale sample of about 200.000 participants. Prior to their participation, respondents made their own site-wide user IDs and passwords, which enabled unique identification and repeated participation in this study. Other than gathering detailed demographics (including political ideology) and session information, the study assigned respondents to one of 95 different attitude domains for the IAT assessment, and followed that with a random selection of one of 20 individual difference measures. These latter measures are our focus. Repeat participations were possible and these were directed to a new domain without replacement (Hussey et al, 2018).

#### Sample

Respondents who are citizens of the United States, residing in the United States, and reported English as their primary language were used in our analysis. These criteria resulted in a retention of 161,058 respondents with complete data on their respective individual difference measures (51.50% of the overall sample *n* = 312,709). Although the criteria result in a drop in sample size, this subsample helps reduce source familiarity bias (for stimulus familiarity, see Malda et al., 2010) by ensuring that self-identified political identity is situated in a single national and language context, and that a potential absence of invariance due to different political contexts or a lack of language proficiency can be ruled out. For each scale, the sample size ranged from 3,733 (Bayesian Racism, Need for Cognitive Closure-Closedmindedness, and Need for Cognitive Closure-Decisiveness) to 3,996 (Rosenberg Self-Esteem). Curran et al. (2016) performed simulations with the psychometric method used in the current study (moderated nonlinear factor analysis [MNLFA] described below), and they found good convergence (99.99%), score recovery, and power with sample sizes from 500 to 2000 and number of Items 6, 12, and 24. The sample size of our data satisfies the conservative estimate of at least 10 observations per estimated parameter in factor analysis (Bentler & Chou, 1987), and will be able to estimate the necessary correlations underlying the analyses with a high degree of precision (Schönbrodt & Perugini, 2013). Similarly, large sample sizes have acceptable Type I error rates in simulation studies (e.g., French & Finch, 2006).

Within this target sample, respondents provided a rating on their political ideology on a 7-point Likert-type scale, ranging from strongly liberal to strongly conservative.

#### Target Measures

The 24 targeted individual difference measures in the data set, including citations to example papers that have tested ideological differences on the measures, are presented in Table 1. We included measures that were available in the data set and that assess constructs that have been tested for ideological differences in the past. We did not select for measures that have previously shown ideological differences or similarities because measurement noninvariance could plausibly affect inferences about both differences and similarities. The measures include those assessing personality traits (e.g., Openness; John & Srivastava, 1999), political values (e.g., right-wing authoritarianism; Altemeyer, 1996), motivational styles (e.g., need for closure; Webster & Kruglanski, 1994), and self-views (e.g., self-esteem; Rosenberg, 1965). When scales include subscales, we assess the subscales individually.

**Table 1.** Scales (in Alphabetical Order) and Number of Items per Scale in the Attitudes 2.0 Data Set That Have Been Tested for Ideological Differences.

Available measures	Measure citation	Example test of ideological differences
Balanced Inventory of Desirable Responding–Impression Management (18 items)	Paulhus (1988)	Wojcik et al. (2015)
Balanced Inventory of Desirable Responding–Self Deception (18 items)	Paulhus (1988)	Wojcik et al. (2015)
Bayesian Racism (16 items)	Uhlmann, Brescoll, and Machery (2010)	Bianchi et al. (2018)
Belief in a Just World (6 items)	Dalbert, Lipkus, Sallay, and Goch (2001)	Kugler et al. (2010)
Big 5 Inventory–Agreeableness (9 items)	John and Srivastava (1999)	Gerber et al. (2010)
Big 5 Inventory–Openness (10 items)	John and Srivastava (1999)	Gerber et al. (2010)
Big 5 Inventory–Extraversion (8 items)	John and Srivastava (1999)	Gerber et al. (2010)
Big 5 Inventory–Conscientiousness (9 items)	John and Srivastava (1999)	Gerber et al. (2010)
Big 5 Inventory–Neuroticism (8 items)	John and Srivastava (1999)	Gerber et al. (2010)
Humanitarianism–Egalitarianism (10 items)	Katz and Hass (1988)	Feinstein et al. (2016)
Need for Cognition (18 items)	Cacioppo, Petty, & Kao (1984)	Stern et al. (2013)
Need for Cognitive Closure–Order (10 items)	Webster and Kruglanski (1994)	Ksiazkiewicz et al. (2016)
Need for Cognitive Closure–Ambiguity (9 items)	Webster and Kruglanski (1994)	Ksiazkiewicz et al. (2016)
Need for Cognitive Closure–Predictability (8 items)	Webster and Kruglanski (1994)	Ksiazkiewicz et al. (2016)
Need for Cognitive Closure–Decisiveness (7 items)	Webster and Kruglanski (1994)	Ksiazkiewicz et al. (2016)
Need for Cognitive Closure–Closed-mindedness (8 items)	Webster and Kruglanski (1994)	Ksiazkiewicz et al. (2016)
Personal Need for Structure (12 items)	Neuberg and Newsom (1993)	Krosch et al. (2013)
Protestant Ethic (11 items)	Katz and Hass (1988)	Schlenker et al. (2012)
Ring-Wing Authoritarianism (20 items)	Altemeyer (1996)	Wilson and Sibley (2013)
Rosenberg Self-Esteem (10 items)	Rosenberg (1965)	Onraet et al. (2013)
Self-Monitoring (18 items)	Snyder (1987)	Berinsky (2004)
Social Dominance Orientation (12 items)	Pratto et al. (1994)	Pratto et al. (1994)
Spheres of Control–Interpersonal Control (10 items)	Paulhus (1983)	Harell et al. (2017)
Spheres of Control–Personal Efficacy (10 items)	Paulhus (1983)	Harell et al. (2017)

### Netherlands Data Set

The LISS (Longitudinal Internet Studies for the Social Sciences) panel is an ongoing and online representative sample of the Netherlands administered by CentERdata (Tilburg University, The Netherlands). Panel members are Dutch individuals who participate in monthly internet surveys. The panel is based on a true probability sample of households drawn from the population register by Statistics Netherlands. Households that could not otherwise participate are provided with a computer and internet connection.

#### Sample

We focus on participants who completed the first waves of the “Politics and Values” and “Personality” core studies. These two studies include multiple measures relevant for the psychology of ideological differences. Moreover, because we are using the first wave, we can be sure that the responses are not contaminated with practice effects. CentERdata provided information on the sample sizes of the two data collections (*n* = 6,808 for the Wave 1 Personality core study, and *n* = 6,811 for the Wave 1 Politics and Values core study), and based on the response rates (~75%-80%), the consolidated sample should have above approximately 5,000 participants. This was the case. In the main analysis, the final sample size in the consolidated data set ranged from 5,092 (Need for Cognition) to 5,111 (the Big Five measures and Satisfaction with Life). We had sufficient power for the planned analysis (i.e., at least 10 observations per estimated parameters in a factor model; Bentler & Chou, 1987) and were able to estimate the necessary correlations within the same model with a high degree of precision.

Within this target sample, respondents reported on their political ideology on a 11-point Likert-type scale. Only the extreme points (0 and 10) had labels (“left” and “right”).

#### Target Measures

The 11 targeted individual difference measures in the data set, including citations to example papers that have tested ideological differences on the measures are presented in Table 2. Similar to the U. S. data set, we included measures that were available in the data set and that assess constructs that have been tested for ideological differences (and similarities) in the past. The measures include those assessing personality traits (e.g., Openness; Goldberg, 1999), motivational styles (e.g., need for cognition; Cacioppo & Petty, 1984), and self-views (e.g., self-esteem; Rosenberg, 1965). When scales include subscales, we assess the subscales individually.

**Table 2.** Scales (in Alphabetical Order) and Number of Items per Scale in the LISS Panel Data Set That Have Been Tested for Ideological Differences.

Available measures	Measure citation	Example test of ideological differences
Big 5 Inventory–Agreeableness (10 items)	Goldberg (1999)	Gerber et al. (2010)
Big 5 Inventory–Openness (10 items)	Goldberg (1999)	Gerber et al. (2010)
Big 5 Inventory–Extraversion (10 items)	Goldberg (1999)	Gerber et al. (2010)
Big 5 Inventory–Conscientiousness (10 items)	Goldberg (1999)	Gerber et al. (2010)
Big 5 Inventory–Neuroticism (10 items)	Goldberg (1999)	Gerber et al. (2010)
Need for Cognition (18 items)	Cacioppo, Petty, & Kao (1984)	Stern et al. (2013)
Need to Evaluate (16 items)	Jarvis and Petty (1996)	Federico and Schneider (2007)
Positive and Negative Affect Scale–Positive affect (10 items)	Watson et al. (1988)	Onraet et al. (2013)
Positive and Negative Affect Scale–Negative affect (10 items)	Watson et al. (1988)	Onraet et al. (2013)
Rosenberg Self-Esteem (10 items)	Rosenberg (1965)	Onraet et al. (2013)
Satisfaction with Life (5 items)	Diener et al. (1985)	Onraet et al. (2013)

*Note*. LISS = Longitudinal Internet Studies for the Social Sciences.

### Analysis Strategy: Moderated Nonlinear Factor Analysis

We test measurement invariance using MNLFA. MNLFA provides a generic method to test for measurement invariance and DIF by combining the rigor and flexibility of multigroup confirmatory factor analysis and the multiple-indicator multiple-cause model, where the effects of multiple categorical and continuous covariates (i.e., grouping variables) on the measurement can be assessed (Bauer, 2017). In MNLFA, DIF (measurement noninvariance) is evaluated as a form of parameter moderation.

In the case of a unidimensional construct, a factor model is specified as a latent factor measured by multiple items, and one or more exogenous variables (in our case political ideology and its quadratic term as ideological extremity) can alter any subset of model parameters in the measurement model. These parameters include the mean and variance of the latent factor, and the factor loading, item intercept, and residual variance for specific items. If the moderation effect of the exogenous variables is restricted to the first subset of parameters (i.e., factor mean and variance), measurement invariance is assumed, whereas if the moderation effect is found to be present for the item parameters (i.e., factor loadings and item intercepts), DIF is detected and measurement invariance is not tenable.

Specifically, based on previous research on these psychological measures, we assume that items on the psychological construct have nonzero loadings (i.e., configural invariance assumed). We include political ideology and ideological extremity as covariates in the measurement model predicting the mean and variance of the factor scores, as well as the item factor loadings and intercepts. Responses on the political ideology item were linearly transformed to have a range of 0 to 1, with 0 indicating strongly liberal (left) and 1 indicating strongly conservative (right). Ideological extremity was computed as the quadratic term of the centered score of ideology. To create this quadratic term, we first transformed political identity to have a range of −1 to 1 and then used a quadratic transformation. This sequence of steps ensures that our measures of both political ideology and ideological extremity have a range from 0 to 1. These transformations aim to provide a straightforward interpretation on the size of DIF and how strongly political ideology and ideological extremity are related to the target construct.

In technical terms, factor scores in this model are a function of an overall intercept plus a weighted linear combination of the two covariates (Gottfredson et al., 2019). Factor variances are conditional on an intercept multiplied by an exponential function of the covariates (the exponential function is used to avoid negative variance values). Factor loadings and intercepts are each a function of an overall mean loading and intercept, plus a linear combination of the covariates. Coding for testing invariance is available at this link: https://osf.io/eypfh/?view_only=d80cf61916254585a052e014471abaa0.

### Procedures to Test Measurement Invariance

The measurement invariance and DIF of each scale/subscale in each data set are tested with MNLFA with the R package aMNLFA (Gottfredson et al., 2019) and MplusAutomation (Hallquist & Wiley, 2018), in combination with M*plus*7.3 (Muthén & Muthén, 1998-2012).

Following Curran et al (2014) and Gottfredson et al (2019), we conduct the analysis for each scale/subscale in four steps.

Step 1 involves the specification and analysis of a baseline model. This is the initial factor model with political ideology and extremity to predict the mean and variance of the latent factor only. In this model, an alpha level of .10 was recommended to retain any potential effects of ideology and extremity on the means and the variance (Gottfredson et al., 2019).

Step 2 involves the initial DIF assessment. As typical in the DIF literature, the test of DIF is performed for one item at a time, while using all other items, which are assumed to be invariant, as anchor items. The covariates’ effects are specified on the factor loading and item intercept. At this step, an alpha level of .05 for retaining predictor effects on factor loadings was recommended. Once a factor loading effect is retained, its effect on the item intercept is also automatically retained (which is a routine procedure to include the main effect if the interaction effect is included in moderation analysis). Effectively, the total number of models that are run in this step is equal to the number of items of the scale.

Step 3 tests the model with all significant DIF effects flagged in the previous step simultaneously. This aims to form the final scoring model that accounts for DIF and impact on the factor mean. The Benjamini–Hochberg family-wise error correction (Thissen et al., 2002) to the loading DIF parameters are applied to multiple significance tests for DIF parameters, and the same correction is applied to the non-DIF intercept parameters as well (Gottfredson et al., 2019).

Step 4 tests the final model with all effects identified in Step 3 to obtain parameter estimates for the DIF and impact effects and generates the factor scores with this impact accounted for. In cases where the impact of political ideology and/or extremity on the latent factor was dropped due to their nonsignificant effects in the previous step, these effects were added back to the final model to facilitate comparisons of impact before and after DIF were accounted for. In all models, the full-information maximum likelihood estimation is used to account for missing data.

We compare estimates of ideological differences on the included scales before and after accounting for DIF effects. The baseline model in Step1 enables us to estimate ideological differences before accounting for potential DIFs (i.e., the regression coefficient from the ideology to the latent factor). The final model in Step 4 provides estimates of the ideological differences after accounting for DIF effects. A comparison of associations based on the unadjusted and adjusted scores helps determine the impact these item biases have on group comparisons.

## Results

### Evaluation of Results: DIF Items

Tables 3 and 4 summarize the overall results of the invariance tests for the United States and Dutch data, respectively. The items flagged as DIF items in Step 2 of the analysis (with an alpha level of .05.) are listed for each scale and the type of DIF (the columns in the table). There are two types of DIF, uniform and nonuniform. Uniform DIF occurs when there are ideological differences in item intercepts after statistically adjusting for ideological differences on the latent variable. When items have uniform DIF, it means that there are group differences on the item regardless of the participants’ level on the construct. Nonuniform DIF occurs when there are differences in the factor loading of the item on the latent construct, that is, there are differences in how the item is related to the latent construct. Uniform and nonuniform DIF can affect an item simultaneously. A list of items indicating whether there was uniform and nonuniform DIF due to political ideology and ideological extremity, respectively, is in the online supplement in Table S1 and Table S2. All DIF had effect sizes *r* < .25 as shown in the tables.

**Table 3.** Summary of DIF Across Ideological Groups in the Attitudes 2.0 Data Set (The United States).

Measure	DIF due to political ideology				DIF due to ideological extremity			
	Uniform	Nonuniform (factor loading differences)	Both uniform and nonuniform	DIF, %	Uniform	Nonuniform (factor loading differences)	Both uniform and nonuniform	% DIF
Social Dominance Orientation	SD1, SD3	SD8	SD4, SD5, SD6, SD7, SD9, SD10. SD11, SD12	92	SD1, SD3, SD4, SD5	SD9	SD7	50
Humanitarianism–Egalitarianism	HE8,HE9		HE1, HE2, HE3, HE4, HE5, HE7, HE10	90	HE5, HE8		HE2	30
Big 5 Inventory–Openness	BFI_O1, BFI_O2, BFI_O3, BFI_O5, BFI_O9, BFI_O10	BFI_O8	BFI_O6, BFI_O7	90	BFI_O6, BFI_O7, BFI_O8	BFI_O2, BFI_O9	BFI_O3	60
Ring-Wing Authoritarianism	RWA4, RWA6, RWA7, RWA8, RWA11, RWA16, RWA17	RWA1, RWA13, RWA14	RWA2, RWA3, RWA5, RWA9, RWA15, RWA20	80	RWA2, RWA7, RWA8, RWA9, RWA12,	RWA4, RWA5, RWA6, RWA11, RWA13, RWA14, RWA17, RWA19, RWA20	RWA1, RWA3	80
Balanced Inventory of Desirable Responding–Impression Management	IM3, IM6, IM13, IM15, IM16, IM18	IM10. IM14	IM1, IM4, IM5,IM11	67	IM11	IM16		11
Protestant Ethic	PE1, PE11	PE6	PE2, PE4,PE5, PE9	64	PE11	—	PE5, PE9	27
Bayesian Racism	BRS4, BRS6, BRS14, BRS15	BRS9, BRS13	BRS2, BRS7, BRS10. BRS11	63	BRS5, BRS10. BRS13	BRS16	—	25
Need for Cognitive Closure–Closed-mindedness	NFCC_C2, NFCC_C3, NFCC_C4, NFCC_C8	—	NFCC_C6	63	NFCC_C1, NFCC_C2, NFCC_C3, NFCC_C4	—	NFCC_C6	63
Rosenberg Self-Esteem	RSE2, RSE6, RSE8, RSE10	RSE3, RSE5	—	60	RSE5, RSE10	—	RSE1, RSE2	40
Need for Cognitive Closure–Ambiguity	NFCC_A6, NFCC_A8	—	NFCC_A3, NFCC_A4 NFCC_A7	56	NFCC_A2, NFCC_A4, NFCC_A5, NFCC_A6, NFCC_A9		—	56
Balanced Inventory of Desirable Responding–Self Deception	SDE1, SDE5, SDE6, SDE7, SDE12, SDE13, SDE14, SDE15, SE16	SDE10	—	50	SDE6, SDE9, SDE11, SDE12, SDE13, SDE14, SDE16, SDE18		—	44
Belief in a Just World	BJW4	—	BJW1, BJW5	50	BJW2, BJW4, BJW5, BJW6	—	—	67
Big 5 Inventory–Extraversion	BFI_E2, BFI_E3, BFI_E6, BFI_E7	—	—	50	BFI_E5, BFI_E6, BFI_E8	—	BFI_E2	50
Need for Cognition	NFC1, NFC2, NFC14, NFC18	NFC3, NFC4, NFC13	NFC5, NFC12	50	NFC3, NFC15	NFC7	—	17
Need for Cognitive Closure–Order	NFCC_O1, NFCC_O5, NFCC_O6	NFCC_O7, NFCC_O9	—	50	NFCC_O1, NFCC_O5	—	—	20
Personal Need for Structure	PNS2, PNS3, PNS6, PNS10. PSN12	—	—	42	PNS10	—	—	8
Spheres of Control–Personal Efficacy	SOC_PE7, SOC_PE8, SOC_PE9	—	SOC_PE2	40	SOC_PE3,SOC_PE10	SOC_PE6	SOC_PE1 SOC_PE5, SOC_PE9	60
Big 5 Inventory–Neuroticism	BFI_N1, BFI_N2	BFI_N3	—	38	BFI_N8	BFI_N3	—	25
Self-Monitoring	SM7, SM8, SM10. SM12, SM16	—	SM3	33	SM4, SM7, SM11, SM14, SM16	SM9	—	33
Need for Cognitive Closure–Predictability	NFCC_P1, NFCC_P5	—	—	25	NFCC_P1	—	—	13
Big 5 Inventory–Conscientiousness	BFI_C5	BFI_C3	—	22	BFI_C1, BFI_C4	BFI_C6	—	33
Spheres of Control–Interpersonal Control	SOC_IC2	SOC_IC9	—	20	SOC_IC5, SOC_IC8	—	—	20
Need for Cognitive Closure–Decisiveness	NFCC_D2	—	—	14	NFCC_D1	NFCC_D3, NFCC_D5	—	43
Big 5 Inventory–Agreeableness	—	—	—	0	BFI-A5	—	—	10

*Note*. Rows are sorted by the percentage of items with DIF due to political ideology. DIF = differential item functioning.

**Table 4.** Summary of DIF Across Ideological Groups in the LISS Panel (The Netherlands).

Measure	DIF due to political ideology				DIF due to ideological extremity			
	Uniform (intercept differences)	Nonuniform (factor loading differences)	Both uniform and nonuniform	DIF, %	Uniform (intercept differences)	Nonuniform (factor loading differences)	Both uniform and nonuniform	% DIF
Need to Evaluate	A085, A087, A088, A089, A094, A095, A096, A097, A098	A086, A091, A093	—	75	A084, A085, A094	A087, A096	A090	38
Need for Cognition	A167, A168, A170. A172, A173, A177, A181	—	A175, A179, A180. A183	61	A167, A171, A174, A180. A183	A178	—	33
Big 5 Inventory–Extraversion	A020. A030. A045, A055	—	A050	50	A035, A060	—	—	20
Big 5 Inventory–Conscientiousness	A037, A042, A052, A062	—	A057	50	A022, A037	A042	A057	40
Rosenberg Self-Esteem	A072, A073, A075	—	A070. A071	50	A073	A075	A071	30
Big 5 Inventory–Agreeableness	A041, A066	A036	A031	40	A066	A036, A051	—	30
Big 5 Inventory–Openness	A029, A044, A059	—	A039	40	A044, A054, A069	A059	—	40
Positive and Negative Affect Scale–Negative affect	A156	—	A152, A153, A158	40	—	A147, A149, A165	A151	40
Satisfaction with Life	A016	—	A014	40	A018	—	—	20
Big 5 Inventory–Neuroticism	A063	—	A038, A068	30	A033, A043, A063	—	A048	40
Positive and Negative Affect Scale–Positive affect	A148, A155, A159	—	—	30	—	A150	—	10

*Note*. Rows are sorted by the percentage of items with DIF due to political ideology. DIF = differential item functioning; LISS = Longitudinal Internet Studies for the Social Sciences.

Tables 3 and 4 show that DIF is prevalent in all scales in both countries. *There is not a single scale that does not have DIF as a function of political ideology and/or ideological extremity*. The proportion of DIF items in the scales ranged from 0% (Agreeableness in the United States does not have DIF as a function of political ideology) to 92% (Social Dominance Orientation in the United States has almost all items flagged due to political ideology differences). For both data sets, there were typically more DIF items due to political ideology (50% in the United States data and 37% in the Dutch data, respectively) than ideological extremity (37% in the United States data and 31% in the Dutch data, respectively). Moreover, DIF effects due to political ideology seemed to be larger than ideological extremity.

**Figure 1.**
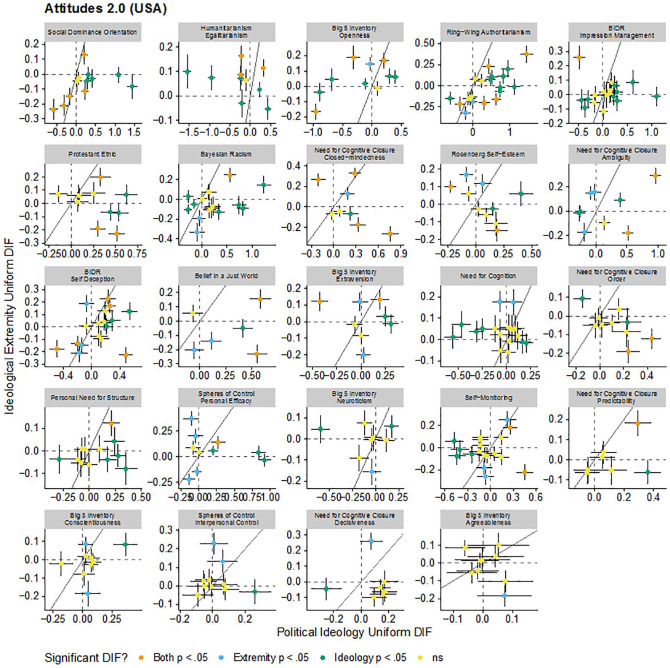
Comparison of estimated uniform DIF for each item for each scale due to political ideology and ideological extremity in the Attitudes 2.0 data set (The United States). *Note*. Dashed vertical and horizontal lines indicate no DIF. Solid line indicates equal DIF for both political ideology and ideological extremity. Error bars are standard errors. DIF = differential item functioning.

**Figure 2.**
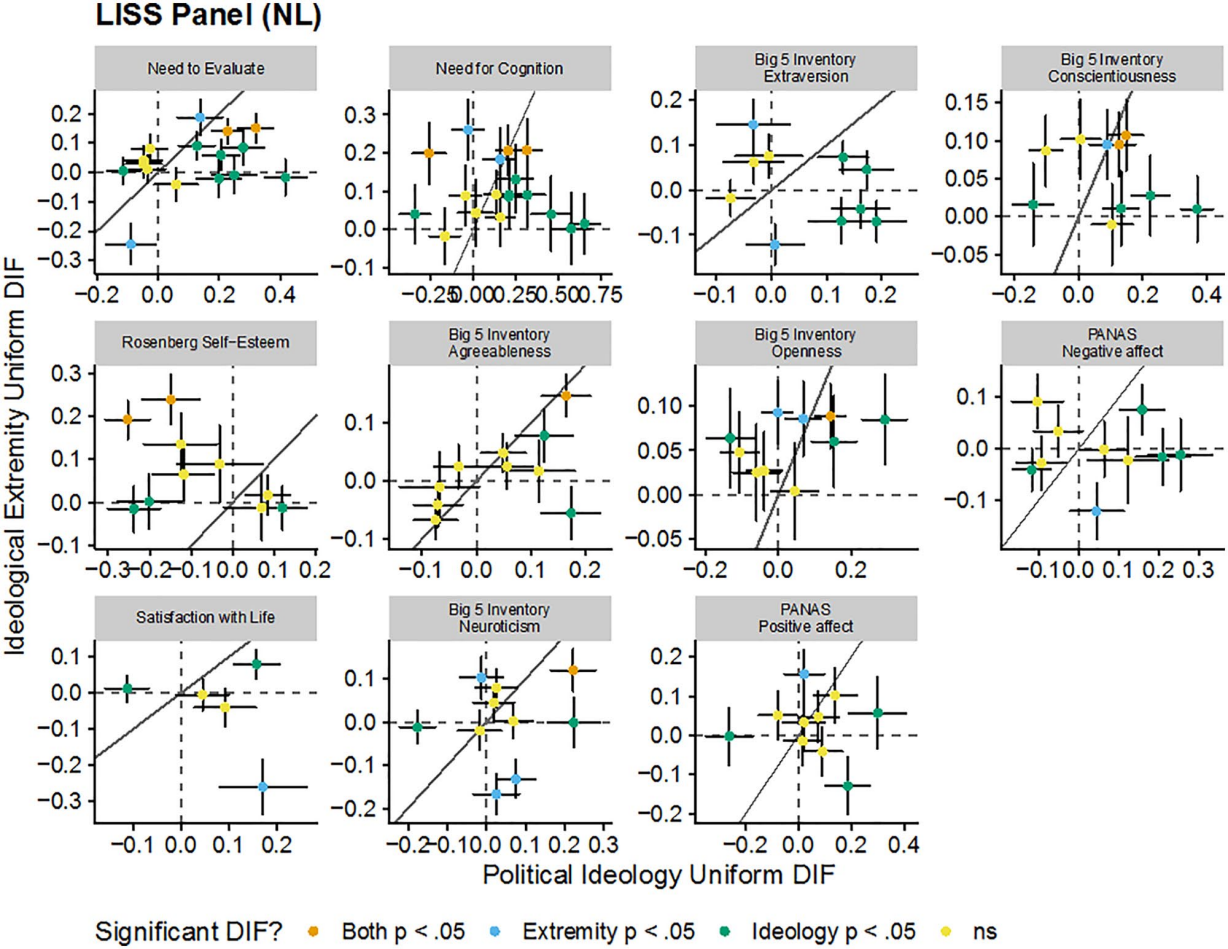
Comparison of estimated uniform DIF for each item for each scale due to political ideology and ideological extremity in the LISS Panel (The Netherlands). *Note*. Dashed vertical and horizontal lines indicate no DFI. Solid line indicates equal DIF for both political ideology and ideological extremity. Error bars are standard errors. DIF = differential item functioning; LISS = Longitudinal Internet Studies for the Social Sciences.

**Figure 3.**
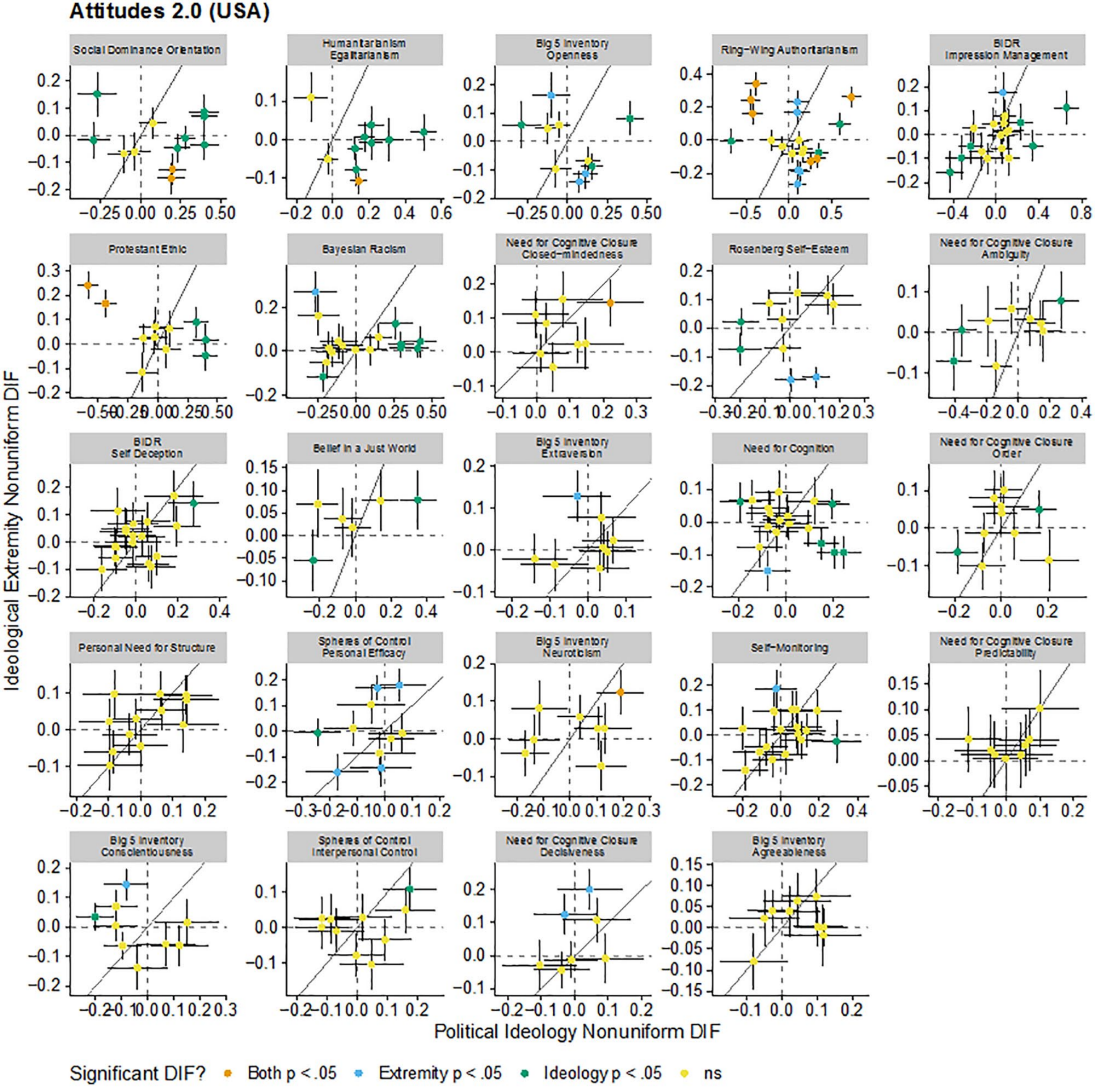
Comparison of estimated nonuniform DIF for each item for each scale due to political ideology and ideological extremity in the Attitudes 2.0 data set (The United States). *Note*. Dashed vertical and horizontal lines indicate no DIF. Solid line indicates equal DIF for both political ideology and ideological extremity. Error bars are standard errors. DIF = differential item functioning.

These patterns are visualized in Figure 1, 2, 3, and 4. Each figure includes the uniform (intercept) or nonuniform (factor loading) DIF estimates for political ideology and ideological extremity for each item in each scale in each sample. Each panel in the figure is a scale and each point in each panel is a scale item. Points further to the right on the *x*-axis have greater political ideology DIF and points higher on the *y*-axis have great ideological extremity DIF. The dashed vertical and horizontal lines indicate no DIF and the solid diagonal line indicates equal political ideology and ideological extremity DIF. There did not seem to be consistency in DIF as functions of political ideology and ideological extremity: for some scales, effects of political ideology and ideological extremity tended to converge (e.g., the same set of uniform DIF items were identified for the social dominance orientation scale for both political ideology and ideological extremity), whereas in some other cases different sets of items were flagged. These DIF effects went both directions and may have attenuated or canceled out overall DIF effects at scale level.

**Figure 4.**
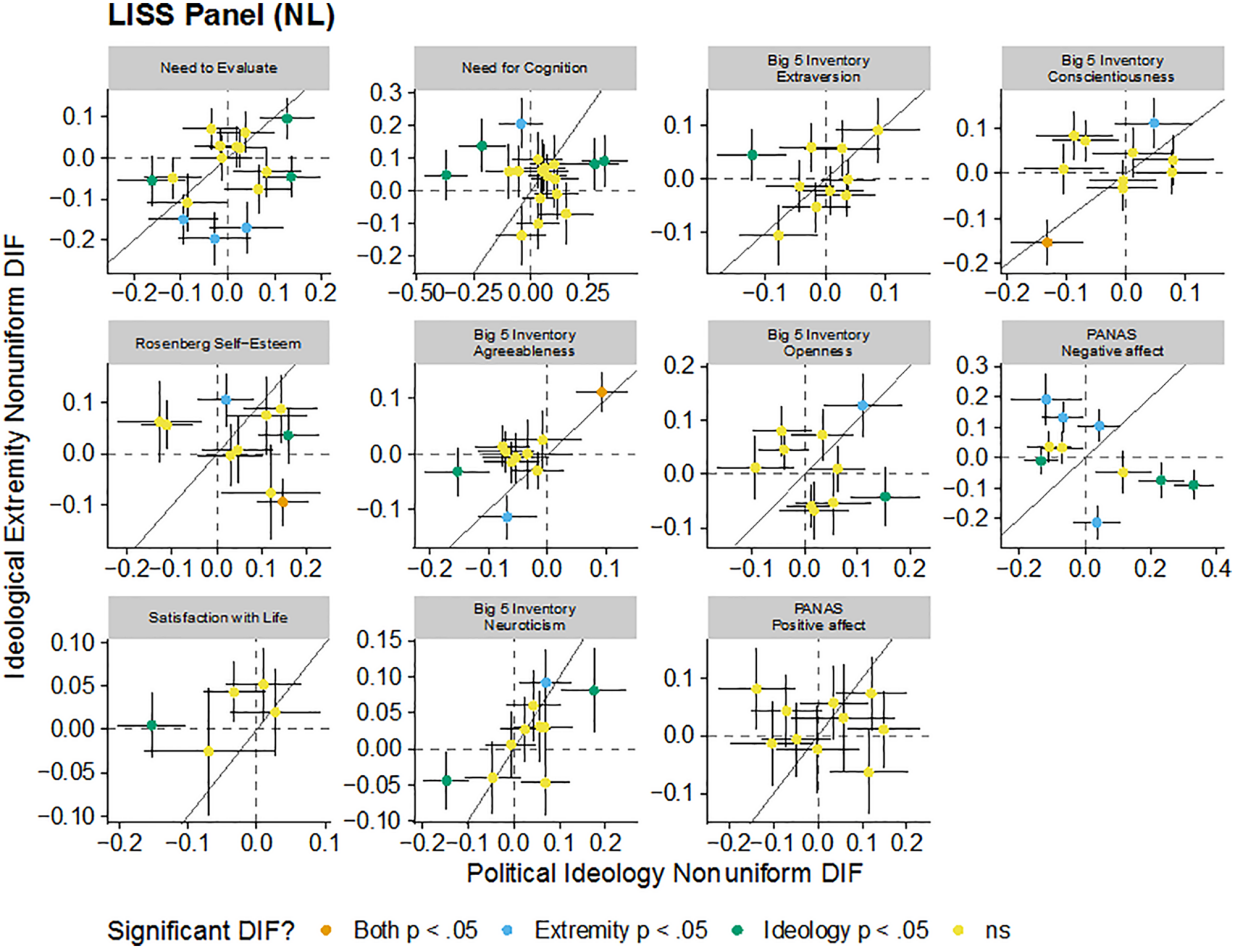
Comparison of estimated nonuniform DIF for each item for each scale due to political ideology and ideological extremity in the LISS Panel (The Netherlands). *Note*. Dashed vertical and horizontal lines indicate no DFI. Solid line indicates equal DIF for both political ideology and ideological extremity. Error bars are standard errors. DIF = differential item functioning; LISS = Longitudinal Internet Studies for the Social Sciences.

Tables 3 and 4 also show that there are more items with uniform DIF than with nonuniform DIF (except for a very few cases, e.g., the Negative Affect scale in the Dutch data had more nonuniform than uniform DIF items). This suggests that the primary issue facing items are different item intercepts, although nearly every comparison had a least one item with nonuniform DIF indicating that there are differences in the factor structure of the constructs across ideological groups.

Since there was variation in the amount and effect sizes of DIF for each comparison, a split of 50% of DIF items due to political ideology was applied in summarizing the results. Nine scales out of 24 in the United States (Social Dominance Orientation, Humanitarianism-Egalitarianism, Openness, Ring-Wing Authoritarianism, Impression Management, Protestant Ethic, Bayesian Racism, Need for Cognitive Closure–Closed-mindedness, Rosenberg Self-Esteem, and Need for Cognitive Closure–Ambiguity), and two out of 11 scales in the Netherlands (Need to Evaluate and Need for Cognition) had DIF due to political ideology on over 50% of items. These DIF prevalent scales were both directly politically relevant (e.g., Social Dominance Orientation and Ring-Wing Authoritarianism) and indirectly relevant to politics (e.g., Impression Management, Openness, Need for Cognition, and Self-Esteem). This highlights that measurement noninvariance across ideological comparisons is not just a problem for research on politically relevant variables but also for constructs without less direct connections to politics.

For constructs measured in both data sets, Need for Cognition, Openness, and Rosenberg Self-Esteem showed more DIF in the United States than in the Netherlands. Extraversion had similar levels of DIF in both countries. Conscientiousness, Neuroticism, and Agreeableness showed fewer DIF in the United States than in the Netherlands. Agreeableness in the United States (as measured with John & Srivastava, 1999) had no DIF for differences in political ideology. This is not because Agreeableness as a construct is free from DIF. In the United States, Agreeableness has one item with uniform DIF for differences in ideological extremity (Table 3). Moreover, Agreeableness in the Netherlands (as measured with Goldberg, 1999), had DIF on four items for political ideology and three items for ideological extremity (Table 4). This points to potential cultural differences in how these scales are interpreted and used by people with different political ideologies. It may also point to differences between the two operationalizations of Agreeableness and their susceptibility to measurement noninvariance.

### Evaluation of Ideological Comparisons

**Figure 5.**
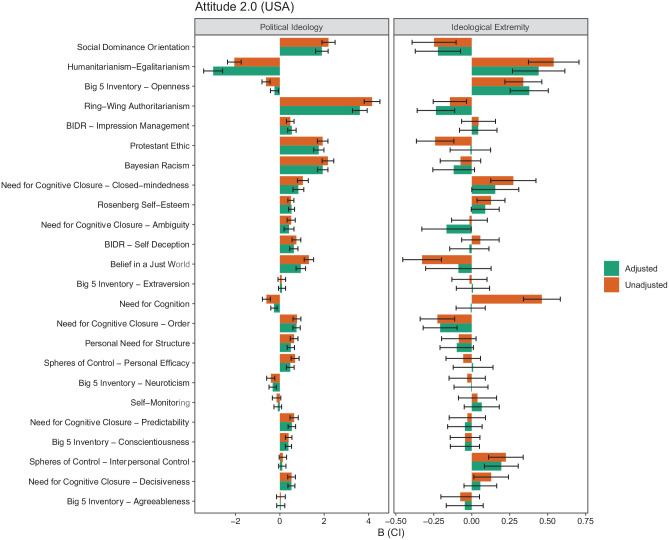
Comparison of political ideology’s and ideological extremity’s association with scales unadjusted and adjusted for measurement invariance in the Attitudes 2.0 data set (The United States). *Note*. Rows are sorted by the percentage of items with DIF due to political ideology. DIF = differential item functioning.

**Figure 6.**
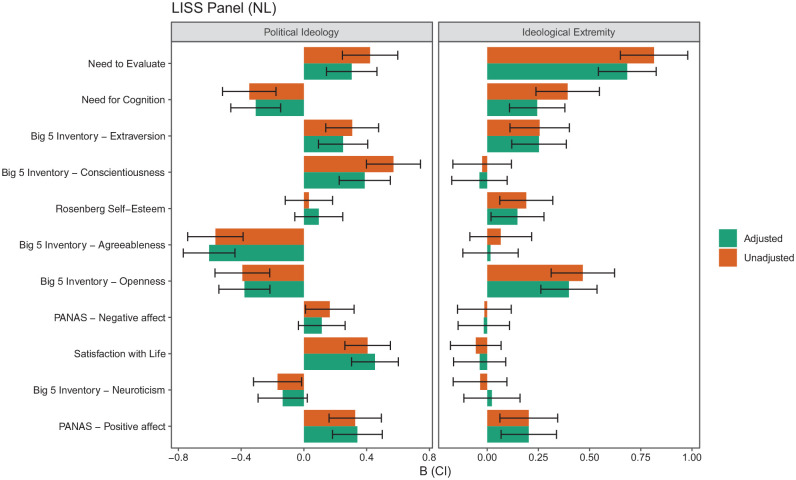
Comparison of political ideology’s and ideological extremity’s association with scales unadjusted and adjusted for measurement invariance in the LISS Panel (The Netherlands). *Note*. Rows are sorted by the percentage of items with DIF due to political ideology. DIF = differential item functioning; LISS = Longitudinal Internet Studies for the Social Sciences.

We assess how correcting for DIF affects the assessment of ideological differences. We do this by comparing the path estimates in the MNLFA models before and after accounting for DIF in the measurement model. Specifically, the path estimates of political ideology and ideological extremity are compared for the same scale of unadjusted (Step 1) and adjusted scores (Step 4) to gauge the (partial) impact of DIF on the mean of the target scale (the impact on variance is simultaneously controlled but is not presented in the main text to maintain focus on our key research question, but this information is in our code and output). We assess if a hypothetical author who used the unadjusted vs. the adjusted scales would come to different conclusions by comparing the direction of the differences and their significance between the unadjusted and adjusted scales. These decisions assume that the hypothetical author uses an alpha level of .05. Summaries of these tests are in the online supplement Tables S4 and S5. They are visualized in Figures 5 and 6.

When first looking at the changes for political ideology in Figures 5 and 6 (left panel; see further details in online supplement Tables S4 and S5), we see that there is substantial variation in the extent to which coefficients are affected by the adjustment. In the United States data, a few rather large effects (B > 1.000) were present, and these were mainly for scales with a majority of DIF items (over 50%). The presence of a large amount of DIF items in a scale suggests that using all other items than the target DIF item as anchor items in DIF detection may challenge the stability and validity of the DIF estimates. Moreover, to account for the DIF effects in the measurement model, the final model incorporated a lot of additional parameters, and caution is needed in interpreting the estimated affect parameters due to possibly unstable solutions. For these scales, the estimated factor loadings tend to be lower than in cases where less than 50% of DIF items are present in a scale, which may artificially boost the impact estimates.

With regard to the impact of political ideology on these scales, the difference between the unadjusted (B = 4.156) and adjusted (B = 3.605) coefficients for right-wing authoritarianism is .551 in the data from the United States, a relatively large change. Other changes are much smaller, such as the difference between the unadjusted (B = 0.399) and adjusted (B = 0.389) coefficients for conscientiousness in the United States data (difference = .01). Differences in the coefficients did not typically translate into different conclusions for a hypothetical researcher using a *p* < .05 decision rule; this only occurred two times and only in the Dutch data (Figure 6). One measure where the corrections changed conclusions is for neuroticism. In the Netherlands, there was a negative association between political ideology and neuroticism (B = −0.168), suggesting that leftists were more neurotic than rightists. However, after adjusting for DIF the estimate was smaller and no longer significant (B = −0.135). Although the difference between the two estimates is relatively small (difference = .033), because the unadjusted effect is already small, the reduced size of the adjusted estimate was sufficient to change the conclusions (based on *p* < .05). Similarly, political ideology was positively related to negative affect before DIF was accounted for (B = 0.165), and the association became nonsignificant after the DIF correction (B = 0.114) in the Netherlands.

A broadly similar pattern of findings emerged for political extremity, as summarized in Figures 5 and 6 (right panels; see further details in the online supplement Tables S6 and S7). There was variation in the size of the difference between the unadjusted and adjusted estimates, but the number of times conclusions changed was relatively small; this occurred seven times in the United States data set and did not occur in the Dutch data. One consequential change was for belief in a just world in the United States data set. The unadjusted coefficient suggests a relatively large effect whereby people at the extreme see the world as less just (B = −0.327); however, the adjusted coefficient suggests that people at the extreme do not significantly differ from their less extreme counterparts (B = −0.088) and the estimate is approximately 1/4th the original size. Similarly, the unadjusted coefficient for the need for cognition in both data sets suggests a relatively large effect whereby people at the extreme have a greater need for cognition (The United States, B = 0.462, The Netherlands, B = 0.393); however, the adjusted coefficient suggests that people at the extreme do not significantly differ from their less extreme counterparts (The United States, B = −0.007) or are not as different from their less extreme counterparts (The Netherlands, B = 0.244).

To further illustrate measurement noninvariance, we look at two scales more closely.

### Deep Dive: Belief in a Just World

In this six-item scale in the United States data set, three items (BJW1 “Justice always prevails over injustice,” BJW4 “In the long run people will be compensated for injustices,” and BJW5 “People get what they deserve”) showed uniform DIF. Conservatives scored higher than liberals on these items irrespective of their individual construct levels. Two items had nonuniform DIF due to ideology. BJW1 (“Justice always prevails over injustice”) showed a higher loading among conservatives than liberals and BJW5 (“People get what they deserve”) had a higher loading among liberals than conservatives. This suggests that these two items are related to the latent belief in a just world construct differently for liberals and conservatives.

The unadjusted impact of ideology (B = 1.31) suggested a stronger endorsement of the belief in a just world among conservatives than liberals. This might be a slight overestimation, given the uniform DIF items favoring conservatives. With these DIF effects accounted for, the adjusted impact (B = 0.946) was smaller than the unadjusted impact, and possibly more valid.

### Deep Dive: Need for Cognition

In this 18-item scale in the United States, there were in total six uniform DIF items due to political ideology, two of which favored conservatives over liberals (NFC1: “I would prefer complex to simple problems” and NFC2: “I like to have the responsibility of handling a situation that requires a lot of thinking”), and four of which favored liberals over conservatives (NFC5: “I try to anticipate and avoid situations where there is likely a chance I will have to think in depth about something.” NFC12: “Learning new ways to think doesn’t excite me very much.” NFC14: “The notion of thinking abstractly is appealing to me,” and NFC18: “I usually end up deliberating about issues even when they do not affect me personally”). There were four nonuniform DIF items showing a higher loading for conservatives than liberals (NFC3: “Thinking is not my idea of fun,” NFC4: “I would rather do something that requires little thought than something that is sure to challenge my thinking abilities,” NFC5: “I try to anticipate and avoid situations where there is likely a chance I will have to think in depth about something” and NFC12: “Learning new ways to think doesn’t excite me very much”) and one item showing a higher loading for liberals than conservatives (NFC13: “I prefer my life to be filled with puzzles that I must solve”).

The unadjusted impact (B = −0.604) suggested a stronger endorsement of need for cognition among liberals than conservatives, which seemed to be a slight overestimation, given that four DIF items favor the former group and only two DIF items favor the latter group. With these DIF effects accounted for, the adjusted impact (B = −0.265) was smaller than the unadjusted impact, and possibly more valid.

## Discussion

Ideological differences in psychology are a key element of political psychology. We contribute to this literature by testing whether various psychological measures are invariant along the dimension of political ideology, and gauge the impact of measurement noninvariance on ideological differences with data sets in the United States and the Netherlands. We employed moderated nonlinear factor analysis to identify items with differential item functioning in each scale/subscale due to political ideology and ideological extremity. Two main findings are outlined here. First, most scales in both data sets suffered from DIF to some degree, regardless of their political relevance. There was huge variation in the amount of DIF for individual scales (from 0% to 92% of the items), where DIF due to political ideology was more prevalent than DIF due to ideological extremity and uniform DIF was more prevalent than nonuniform DIF. Second, the impact of DIF on the association between political ideology and these scales was relatively limited in both data sets in the sense that substantive conclusions did not always change, although coefficients were different before and after DIF correction.

### Differential Item Functioning and Its Impact: Why It Is Useful to Demonstrate Measurement Invariance

There is increasing awareness that it is useful to demonstrate the level of measurement invariance, not only among distinct groups in cross-cultural research, time points in longitudinal research, but also for less distinct comparisons along continuous variables such as political ideology. Psychometric tools abound to check whether the structure of the construct, item factor loadings, and intercepts are equal, but these are so far only used sporadically rather than consistently in many social science domains (Boer et al., 2018). MNLFA provides a generic framework to check measurement invariance and account for measurement noninvariance with multiple continuous or categorical covariates (Bauer, 2017).

Using MNLFA, we demonstrated variation in the amount and impact of DIF due to both political ideology and ideological extremity among 28 constructs in two data sets. Detailed extrapolations of sources of DIF are hard to achieve. Nonetheless, we observe that scales in which DIF is prevalent seem to share some common characteristics: they are generally long (over 10 items), formulated with more complex wordings (i.e., both positive and negative), and responses are more skewed (e.g., estimated item intercepts tend to be quite far away from the midpoint of the response scale) in comparison to scales with below 50% of DIF items. For example, the Need for Cognition scale has 18-items and includes items with complex wordings and conditional statements; these factor may have all contributed to DIF for this scale’s items. In general, the items in many scales differ markedly from the general guidelines of the International Test Commission (2017, see also van de Vijver & Hambeleton, 1996), which has laid out best practices for item formulation, adaptation, and assessment to retain comparability, particularly in cross-cultural application and after translations (see van de Vijver & Leung, 1997, van de Vijver & Poortinga, 2005). Long, complex, or even multibarreled items (here: both positive and negative wording) are generally considered problematic. Although adjusting for DIF does not always change the estimates of ideological differences on these scales, accounting for DIF provides more valid estimates than ignoring the measurement noninvariance in these scales. Measurement invariance should not be assumed, but rather be tested empirically and with DIF being factored in estimating group differences.

Because we found DIF across a number of scales, it raises the question as to whether this DIF needs to be a worry for researchers and practitioners. On the one hand, because adjusting for DIF did not typically change the global estimates or conclusions about ideological differences and because many of the DIF effect sizes were relatively small, it appears that researchers and practitioners do not need to worry about DIF in most research or clinical settings. On the other hand, there are reasons to still be concerned with possible DIF. First, we did find some places where DIF did change conclusions substantially (e.g., political extremity and belief in a just world). Without testing for DIF, researchers do not know if they are in situation where DIF is not consequential or if they are in a situation where DIF substantially changes the estimates. Second, correcting for DIF often decreased the size of the ideological or extremity difference, suggesting that over the course of many studies unadjusted estimates may overestimate the size of these differences. Third, a related issue is that when adjusting for DIF did change conclusions, it was always a situation where a significant effect became nonsignificant. Given well known tendencies for researchers and journals to publish significant effects, failing to account for DIF might result in more Type I errors in being published and persisting in the literature (e.g., Ferguson & Heene, 2012). Fourth, we did find relatively larger DIF on some individual items. If researchers pick a subset of items from a scale (e.g., due to space constraints) the effect of DIF on conclusions could be larger depending on the precise items chosen. In short, our findings suggest that in many situations adjusting for DIF will not substantially alter researchers’ conclusions; however, when aggregating over many studies or focusing on only a few issues, the impact of DIF may be more acutely felt.

### Robustness, National Similarities, and National Differences in Ideological Differences

Some scales were available in both samples. This allows us to examine ideological differences in both countries. Specifically, in both countries with both unadjusted and DIF adjusted measurements, openness and need for cognition were lower, and conscientiousness was higher among conservatives (rightists) than liberals (leftists). These significant differences seem to be robust and are consistent with prior work (e.g., Jost et al., 2003, among others). However, self-esteem was found to be positively related to being conservative in the United States data (with or without DIF adjustment), while the effect was negative in the Dutch data. Neuroticism showed a negative association with being conservative in the United States data, while a similar negative effect disappeared in the Netherlands after DIF was adjusted. These different patterns potentially point to culture-specifics in ideological differences or scale use.

### Limitations and Future Directions

We could only gauge the effect sizes of DIF with the regression coefficient of the item on the latent factor and the *r* value based on *t* test. This is a limitation of MNLFA. More straightforward effect size measures in such models, as well as effect size benchmarks, should be developed to inform which DIF items exert the most impact. We used two empirical data sets in which the true ideological differences on these scales are unknown, thus it is possible that the DIF unadjusted scores are more valid than the adjusted scores. Testing which measures have the best predictive validity of relevant behaviors may help identify the most valid measurement procedure in future work. This notwithstanding, the empirical demonstration of the presence of DIF draws attention to the potential problem of using observed scale scores or the latent factor scores in a measurement model without accounting for DIF. We flagged all uniform and nonuniform DIF items (presented in the online supplemental materials), and further text analysis and mixed-methods studies can be conducted to uncover the root cause of DIF (Benitez & Padilla 2014). This will help develop assessment methods to become more equivalent across both groups and continuous dimensions.

Our approach for assessing ideological difference (including DIF) was to only focus on ideology and extremity. This raises the question as to whether other variables often associated with ideology, such as age, ethnicity, gender, and education, might be responsible for the DIF effects. This is certainly possible and additional investigations can probe these factors as well as other possible mechanisms behind the DIF effects. We chose to focus on ideology and extremity and to the exclusion of these other demographic variables because many of the demonstrations of psychological differences in ideology do not adjust for demographic factors (e.g., see the meta-analyses Jost et al., 2003; Jost, Sterling, et al., 2017; Jost, Stern, et al., 2017). This helped make our analyses more consistent with the work we were building on, while we point out that this is an important direction for subsequent research.

It is also possible that although our data describe the time and place they were collected, the findings would change in other political eras. A strength of our study was that we examined data from two political systems. Because we consistently found DIF in two different countries with different salient political issues and structures to their political belief systems, we would expect the finding that there is DIF to be consistent. We are less certain that we would find DIF on the same items in different political circumstances. This is because we did not always find that the same items had DIF in the U.S. and the Netherlands samples, suggesting that the different circumstances might make a difference. We also know that the correlations between political ideology and other individual differences can shift depending on the context (Malka et al., 2014). There is no reason to believe that a shifting context could not also shift which items have DIF. This further highlights the utility of assessing DIF in future work.

## Conclusions

We found that scales used to identify ideological differences and similarities are not measurement invariant across political ideology and ideological extremity. Correcting for these differences changes the precise size of the ideological differences, however, this rarely would change the conclusion of a researcher using an alpha of *p* < .05 to study ideological differences. This can give confidence in the ideological similarities and differences reported in the literature. One reason for the relative lack of practical consequences appears to be that items’ DIFs within a scale went in both directions (e.g., liberal and conservative, moderate, and extreme) cancelling out large effects of DIF. Future scale development work should use best practices for scale construction and item wording to minimize measurement noninvariance.

## Supplemental Material

sj-pdf-1-asm-10.1177_1073191120983891 – Supplemental material for Registered Report: Testing Ideological Asymmetries in Measurement Invariance Click here for additional data file. Supplemental material, sj-pdf-1-asm-10.1177_1073191120983891 for Registered Report: Testing Ideological Asymmetries in Measurement Invariance by Mark J. Brandt, Jia He and Michael Bender in Assessment
