# Supplementary material for: Registered Report: Testing Ideological Asymmetries in Measurement Invariance
Source: Assessment. 2021 Jan 29;28(3):687–708. doi: 10.1177/1073191120983891 (PMC7961747; doi:10.1177/1073191120983891)
Supplement: sj-pdf-1-asm-10.1177_1073191120983891 – Supplemental material for Registered Report: Testing Ideological Asymmetries in Measurement Invariance [file sj-pdf-1-asm-10.1177_1073191120983891.pdf]

Table S1

Uniform and Nonuniform Differential Item Functioning for ideology and extremity of items in the Attitudes 2.0 dataset, listed per scale

Scale	ideology				extremity			
	Uniform		Nonuniform		Uniform		Nonuniform	
	sig	r	sig	r	sig	r	sig	r
**Balanced Inventory of Desirable Responding - Impression Management (18-items)**								
*BIDR-IM1: I sometimes tell lies if I have to.	x	0.038	x	0.055		0.003		0.012
BIDR-IM2: I never cover up my mistakes.		0.002		0.013		0.030		0.019
*BIDR-IM3: There have been occasions when I have taken advantage of someone.	x	0.053		0.013		0.008		0.024
BIDR-IM4: I never swear.	x	0.173	x	0.095		0.003		0.025
*BIDR-IM5: I sometimes try to get even rather than forgive and forget.	x	0.067	x	0.033		0.008		0.011
BIDR-IM6: I always obey laws, even if I’m unlikely to get caught.	x	0.045		0.031		0.010		0.006
*BIDR-IM7: I have said something bad about a friend behind his or her back.		0.028		0.018		0.013		0.022
BIDR-IM8: When I hear people talking privately, I avoid listening.		0.022		0.011		0.002		0.010
*BIDR-IM9: I have received too much change from a salesperson without telling him or her.		0.020		0.005		0.006		0.001
*BIDR-IM10: When I was young I sometimes stole things.		0.009	x	0.038		0.000		0.017
BIDR-IM11: I have never dropped litter on the street.	x	0.065	x	0.054	x	0.051		0.029
*BIDR-IM12: I sometimes drive faster than the speed limit.		0.028		0.004		0.001		0.010
*BIDR-IM13: I have done things that I don’t tell other people about.	x	0.064		0.009		0.024		0.014
BIDR-IM14: I never take things that don’t belong to me.		0.028	x	0.036		0.020		0.011
*BIDR-IM15: I have taken sick-leave from work or school even though I wasn’t really sick.	x	0.078		0.014		0.017		0.003
BIDR-IM16: I have never damaged a library book or store merchandise without reporting it.	x	0.039		0.008		0.004	x	0.033
*BIDR-IM17: I have some pretty awful habits.		0.003		0.018		0.005		0.015
BIDR-IM18: I don’t gossip about other people’s business.	x	0.038		0.015		0.015		0.002
**Balanced Inventory of Desirable Responding - Self Deception (18-items)**								
BIDR-SDE1: My first impressions of people usually turn out to be right.	x	0.035		0.010		0.008		0.019
*BIDR-SDE2: It would be hard for me to break any of my bad habits.		0.015		0.002		0.007		0.014

BIDR-SDE3: I don't care to know what other people really think of me.		0.020		0.010		0.022	0.019
*BIDR-SDE4: I have not always been honest with myself.		0.012		0.028		0.001	0.013
BIDR-SDE5: I always know why I like things.	x	0.036		0.002		0.001	0.006
*BIDR-SDE6: When my emotions are aroused, it biases my thinking.	x	0.036		0.016	x	0.035	0.013
BIDR-SDE7: Once I've made up my mind, other people can seldom change my opinion.	x	0.080		0.022		0.028	0.021
*BIDR-SDE8: I am not a safe driver when I exceed the speed limit.		0.013		0.002		0.015	0.000
BIDR-SDE9: I am fully in control of my own fate.		0.025		0.011	x	0.044	0.022
*BIDR-SDE10: It's hard for me to shut off a disturbing thought.		0.028	x	0.037		0.028	0.029
BIDR-SDE11: I never regret my decisions.		0.024		0.015	x	0.038	0.004
*BIDR-SDE12: I sometimes lose out on things because I can't make up my mind soon enough.	x	0.033		0.006	x	0.050	0.008
*BIDR-SDE13: My parents were not always fair when they punished me.	x	0.036		0.021		0.010	0.030
BIDR-SDE14: I am a completely rational person.	x	0.082		0.016	x	0.057	0.004
*BIDR-SDE15: I rarely appreciate criticism.	x	0.076		0.007	x	0.041	0.010
BIDR-SDE16: I am very confident of my judgments.	x	0.052		0.019	x	0.051	0.017
BIDR-SDE17: It's all right with me if some people happen to dislike me.		0.027		0.004		0.010	0.005
*BIDR-SDE18: I don't always know the reasons why I do the things I do.		0.011		0.008	x	0.045	0.017
**Bayesian Racism (16-items)**							
BRS1: I would avoid walking through a Hispanic neighborhood at night.		0.019		0.028		0.022	0.011
BRS2: If you want to make accurate predictions, you should use information about a person's ethnic group when deciding if they will perform well.	x	0.053	x	0.064		0.032	0.005
BRS3: I would prefer to send my children to a majority White school than a majority Black school.		0.019		0.026		0.015	0.003
BRS4: If your personal safety is at stake, it's sensible to avoid members of ethnic groups known to behave more aggressively.	x	0.047		0.025		0.028	0.001
*BRS5: I would be equally likely to give a ride to a Hispanic hitchhiker or a White hitchhiker.		0.010		0.011	x	0.059	0.005
BRS6: When the only thing you know about someone is their race, it makes sense to use your knowledge of their racial group to form an impression of them.	x	0.042		0.026		0.008	0.017
BRS7: Law enforcement officers should pay particular attention to those social groups more heavily involved in crime, even if this means focusing on members of particular ethnic groups.	x	0.112	x	0.034		0.020	0.027
*BRS8: Law enforcement officers should act as if members of all racial groups are equally likely to commit crimes.		0.018		0.014		0.017	0.002

BRS9: If you want to make accurate predictions about whether someone will do well at certain jobs, you should use information about their gender.		0.002	x	0.055		0.001		0.009
BRS10: If I agreed with his or her other positions, I would seriously consider voting for a candidate who believes racial profiling by the police should be allowed.	x	0.077	x	0.065	x	0.056		0.011
BRS11: Cab drivers ought to be legally able to avoid picking up blacks.	x	0.032	x	0.096		0.018		0.004
*BRS12: It is always wrong to avoid someone because members of their racial group are more likely to commit violent crimes.		0.023		0.029		0.012		0.029
BRS13: If it will increase profits, it makes sense to use statistics about the performance of different racial groups.		0.006	x	0.037	x	0.039		0.027
*BRS14: It should be against airport policy to allow airport security to search passengers based on their ethnic group—for example, Arabs more so than others.	x	0.141		0.015		0.027		0.009
*BRS15: It should be illegal for drug agents to search Hispanics more often than Whites for drugs.	x	0.084		0.001		0.011		0.001
BRS16: It's irrational to behave as if members of some racial groups are not more likely to threaten or hurt you.		0.020		0.031		0.015	x	0.048
**Belief in a Just World (6-items)**								
BJW1: Justice always prevails over injustice.	x	0.071	x	0.055		0.013		0.020
BJW2: Injustices in all areas of life (e.g., professional, family, politics) are the exception rather than the rule.		0.018		0.011	x	0.035		0.009
BJW3: People try to be fair when making important decisions.		0.012		0.022		0.015		0.019
BJW4: In the long run people will be compensated for injustices.	x	0.092		0.030	x	0.037		0.015
BJW5: People get what they deserve.	x	0.092	x	0.037	x	0.058		0.013
BJW6: Basically, the world is a fair place.		0.009		0.002	x	0.054		0.004
**Big 5 Inventory – Agreeableness (9-items)**								
*BFI-A1: Tends to find fault with others		0.013		0.016		0.027		0.018
BFI-A2: Is helpful and unselfish with others		0.014		0.024		0.030		0.001
*BFI-A3: Starts quarrels with others		0.000		0.008		0.005		0.006
BFI-A4: Has a forgiving nature		0.007		0.004		0.016		0.011
BFI-A5: Is generally trusting		0.013		0.007	x	0.049		0.016
*BFI-A6: Can be cold and aloof		0.008		0.018		0.024		0.004
BFI-A7: Is considerate and kind to almost everyone		0.002		0.029		0.005		0.001
*BFI-A8: Is sometimes rude to others		0.007		0.013		0.010		0.019
BFI-A9: Likes to cooperate with others		0.004		0.006		0.015		0.014

**Big 5 Inventory – Openness (10-items)**								
BFI-O1: Is original, comes up with new ideas	x	0.095		0.012		0.022		0.020
BFI-O2: Is curious about many different things	x	0.034		0.020		0.008	x	0.057
BFI-O3: Is ingenious, a deep thinker	x	0.043		0.023	x	0.054	x	0.034
BFI-O4: Has an active imagination		0.022		0.029		0.005		0.022
BFI-O5: Is inventive	x	0.061		0.025		0.020		0.013
BFI-O6: Values artistic, aesthetic experiences	x	0.184	x	0.070	x	0.050		0.022
*BFI-O7: Prefers work that is routine	x	0.043	x	0.038	x	0.039		0.011
BFI-O8: Likes to reflect, play with ideas		0.009	x	0.036	x	0.053		0.030
*BFI-O9: Has few artistic interests	x	0.099		0.014		0.010	x	0.032
BFI-O10: Is sophisticated in art, music, or literature	x	0.154		0.012		0.010		0.023
**Big 5 Inventory – Extroversion (8-items)**								
BFI-E1: Is talkative		0.013		0.008		0.005		0.002
*BFI-E2: Is reserved	x	0.078		0.005	x	0.033	x	0.034
BFI-E3: Is full of energy	x	0.057		0.026		0.004		0.006
BFI-E4: Generates a lot of enthusiasm		0.000		0.007		0.026		0.013
*BFI-E5: Tends to be quiet		0.002		0.013	x	0.035		0.007
BFI-E6: Has an assertive personality	x	0.034		0.015	x	0.035		0.009
*BFI-E7: Is sometimes shy, inhibited	x	0.044		0.007		0.008		0.020
BFI-E8: Is outgoing, sociable		0.005		0.011	x	0.066		0.001
**Big 5 Inventory – Conscientiousness (9-items)**								
BFI-C1: Does a thorough job		0.006		0.022	x	0.034		0.023
*BFI-C2: Can be somewhat careless		0.002		0.018		0.020		0.014
BFI-C3: Is a reliable worker		0.018	x	0.047		0.011		0.012
*BFI-C4: Tends to be disorganized		0.006		0.020	x	0.042		0.003
*BFI-C5: Tends to be lazy	x	0.060		0.010		0.021		0.013
BFI-C6: Perseveres until the task is finished		0.015		0.016		0.004	x	0.043

BFI-C7: Does things efficiently		0.019		0.024		0.004		0.021
BFI-C8: Makes plans and follows through with them		0.006		0.024		0.006		0.001
*BFI-C9: Is easily distracted		0.029		0.005		0.005		0.030
**Big 5 Inventory – Neuroticism (8-items)**								
BFI-N1: Is depressed, blue	x	0.094		0.018		0.011		0.018
*BFI-N2: Is relaxed, handles stress well	x	0.035		0.007		0.018		0.017
BFI-N3: Can be tense		0.008	x	0.034		0.001	x	0.033
BFI-N4: Worries a lot		0.005		0.020		0.002		0.018
*BFI-N5: Is emotionally stable, not easily upset		0.022		0.031		0.021		0.010
BFI-N6: Can be moody		0.029		0.020		0.023		0.007
*BFI-N7: Remains calm in tense situations		0.022		0.019		0.002		0.008
BFI-N8: Gets nervous easily		0.007		0.023	x	0.040		0.000
**Humanitarianism-Egalitarianism (10-items)**								
HE1: One should be kind to all people.	x	0.099	x	0.054		0.019		0.003
HE2: One should find ways to help others less fortunate than oneself.	x	0.098	x	0.050	x	0.054	x	0.056
HE3: A person should be concerned about the well-being of others.	x	0.074	x	0.045		0.013		0.012
HE4: There should be equality for everyone—because we are all human beings.	x	0.049	x	0.114		0.023		0.006
HE5: Those who are unable to provide for their basic needs should be helped by others.	x	0.055	x	0.049	x	0.063		0.002
HE6: A good society is one in which people feel responsible for one another.		0.026	x	0.033		0.023		0.029
HE7: Everyone should have an equal chance and an equal say in most things.	x	0.038	x	0.062		0.008		0.000
HE8: Acting to protect the rights and interests of other members of the community is a major obligation for all persons.	x	0.056		0.008	x	0.033		0.020
HE9: In dealing with criminals the courts should recognize that many are victims of circumstances.	x	0.237		0.021		0.024		0.027
HE10: Prosperous nations have a moral obligation to share some of their wealth with poor nations.	x	0.188	x	0.047		0.023		0.012
**Need for Cognition (18-items)**								
NFC1: I would prefer complex to simple problems.	x	0.033		0.015		0.003		0.013
NFC2: I like to have the responsibility of handling a situation that requires a lot of thinking.	x	0.048		0.006		0.006		0.002

*NFC3: Thinking is not my idea of fun.		0.015	x	0.045	x	0.059		0.031
*NFC4: I would rather do something that requires little thought than something that is sure to challenge my thinking abilities.		0.013	x	0.046		0.017		0.020
*NFC5: I try to anticipate and avoid situations where there is likely a chance I will have to think in depth about something.	x	0.055	x	0.033		0.017		0.022
NFC6: I find satisfaction in deliberating hard and for long hours.		0.010		0.024		0.013		0.017
*NFC7: I only think as hard as I have to.		0.004		0.014		0.015	x	0.040
*NFC8: I prefer to think about small, daily projects to long-term ones.		0.019		0.001		0.005		0.005
*NFC9: I like tasks that require little thought once I've learned them.		0.015		0.011		0.013		0.007
NFC10: The idea of relying on thought to make my way to the top appeals to me.		0.003		0.003		0.019		0.002
NFC11: I really enjoy a task that involves coming up with new solutions to problems.		0.006		0.018		0.002		0.005
*NFC12: Learning new ways to think doesn't excite me very much.	x	0.070	x	0.053		0.013		0.031
NFC13: I prefer my life to be filled with puzzles that I must solve.		0.012	x	0.036		0.015		0.017
NFC14: The notion of thinking abstractly is appealing to me.	x	0.110		0.018		0.004		0.006
NFC15: I would prefer a task that is intellectual, difficult, and important to one that is somewhat important but does not require much thought.		0.013		0.007	x	0.048		0.008
*NFC16: I feel relief rather than satisfaction after completing a task that required a lot of mental effort.		0.010		0.018		0.005		0.015
*NFC17: It's enough for me that something gets the job done; I don't care how or why it works.		0.021		0.019		0.013		0.020
NFC18: I usually end up deliberating about issues even when they do not affect me personally.	x	0.078		0.005		0.018		0.023
**Need for Cognitive Closure – Order (10-items)**								
NFCC-O1: I think that having clear rules and order at work is essential for success.	x	0.086		0.014	x	0.037		0.004
NFCC-O2: I find that a well ordered life with regular hours suits my temperament.		0.002		0.002		0.003		0.032
NFCC-O3: I hate to change my plans at the last minute.		0.003		0.009		0.011		0.003
*NFCC-O4: My personal space is usually messy and disorganized.		0.030		0.027		0.017		0.017
NFCC-O5: I believe orderliness and organization are among the most important characteristics of a good student.	x	0.041		0.014	x	0.050		0.026
*NFCC-O6: I think that I would learn best in a class that lacks clearly stated objectives and requirements.	x	0.033		0.000		0.007		0.012
NFCC-O7: I find that establishing a consistent routine enables me to enjoy life more.	x	0.032	x	0.035		0.031		0.016
NFCC-O8: I enjoy having a clear and structured mode of life.		0.025		0.000		0.013		0.015

NFCC-O9: I like to have a plan for everything and everything in its place.		0.030	x	0.034		0.010		0.018
*NFCC-O10: I dislike the routine aspects of my work (studies).		0.006		0.004		0.012		0.018
**Need for Cognitive Closure – Ambiguity (9-items)**								
NFCC-A1: I don't like situations that are uncertain.		0.024		0.012		0.026		0.009
*NFCC-A2: I feel comfortable when I don't understand the reason why an event occurred in my life.		0.027		0.023	x	0.036		0.006
NFCC-A3: When I am confused about an important issue, I feel very upset.	x	0.044	x	0.039		0.004		0.018
NFCC-A4: In most social conflicts, I can easily see which side is right and which is wrong.	x	0.159	x	0.061	x	0.073		0.016
NFCC-A5: I like to know what people are thinking all the time.		0.004		0.022	x	0.038		0.001
NFCC-A6: I dislike it when a person's statement could mean many different things.	x	0.096		0.023	x	0.049		0.021
NFCC-A7: It's annoying to listen to someone who cannot seem to make up his or her mind.	x	0.071	x	0.059		0.025		0.002
NFCC-A8: I feel uncomfortable when someone's meaning or intention is unclear to me.	x	0.053		0.027		0.000		0.007
NFCC-A9: I'd rather know bad news than stay in a state of uncertainty.		0.017		0.007	x	0.042		0.015
**Need for Cognitive Closure – Predictability (8-items)**								
*NFCC-P1: I like to have friends who are unpredictable.	x	0.053		0.019	x	0.049		0.011
*NFCC-P2: I enjoy the uncertainty of going into a new situation without knowing what might happen.		0.011		0.010		0.011		0.008
NFCC-P3: When dining out, I like to go to places where I have been before so that I know what to expect.		0.017		0.014		0.012		0.022
*NFCC-P4: I think it is fun to change my plans at the last moment.		0.008		0.007		0.012		0.005
NFCC-P5: I don't like to be with people who are capable of unexpected actions.	x	0.064		0.005		0.016		0.003
NFCC-P6: I prefer to socialize with familiar friends because I know what to expect from them.		0.010		0.008		0.004		0.003
NFCC-P7: I don't like to go into a situation without knowing what I can expect from it.		0.022		0.000		0.014		0.001
NFCC-P8: I dislike unpredictable situations.		0.010		0.013		0.019		0.011
**Need for Cognitive Closure – Decisiveness (7-items)**								
*NFCC-D1: I would describe myself as indecisive.		0.015		0.008	x	0.079		0.013
*NFCC-D2: When I go shopping, I have difficulty deciding exactly what it is I want.	x	0.038		0.013		0.010		0.002
NFCC-D3: When faced with a problem I usually see the one best solution very quickly.		0.031		0.008		0.017	x	0.052
*NFCC-D4: I tend to put off making important decisions until the last possible moment.		0.026		0.015		0.000		0.006

NFCC-D5: I usually make important decisions quickly and confidently.		0.017		0.005		0.027	x	0.033
*NFCC-D6: I tend to struggle with most decisions.		0.029		0.002		0.012		0.004
*NFCC-D7: When trying to solve a problem I often see so many possible options that it's confusing.		0.027		0.011		0.021		0.027
**Need for Cognitive Closure - Closed-mindedness (8-items)**								
*NFCC-CM1: Even after I've made up my mind about something, I am always eager to consider a different opinion.		0.031		0.000	x	0.034		0.027
NFCC-CM2: I dislike questions which could be answered in many different ways.	x	0.106		0.011	x	0.056		0.031
NFCC-CM3: I feel irritated when one person disagrees with what everyone else in a group believes.	x	0.050		0.021	x	0.040		0.005
*NFCC-CM4: When considering most conflict situations, I can usually see how both sides could be right.	x	0.058		0.006	x	0.103		0.024
*NFCC-CM5: When thinking about a problem, I consider as many different opinions on the issue as possible.		0.017		0.025		0.014		0.007
*NFCC-CM6: I prefer interacting with people whose opinions are very different from my own.	x	0.036	x	0.036	x	0.070	x	0.035
*NFCC-CM7: I always see many possible solutions to problems I face.		0.005		0.003		0.022		0.001
NFCC-CM8: I do not usually consult many different options before forming my own view.	x	0.036		0.008		0.016		0.010
**Personal Need for Structure (12-items)**								
PNS1: It upsets me to go into a situation without knowing what I can expect from it.		0.022		0.007		0.011		0.004
*PNS2: I’m not bothered by things that interrupt my daily routine.	x	0.049		0.023		0.010		0.020
PNS3: I enjoy having a clear and structured mode of life.	x	0.059		0.013		0.007		0.016
PNS4: I like to have a place for everything and everything in its place.	x	0.053		0.019		0.018		0.003
*PNS5: I enjoy being spontaneous.		0.014		0.028		0.003		0.028
*PNS6: I find that a well-ordered life with regular hours makes my life tedious.	x	0.041		0.013		0.011		0.024
PNS7: I don’t like situations that are uncertain.		0.000		0.003		0.019		0.009
PNS8: I hate to change my plans at the last minute.		0.013		0.015		0.013		0.023
PNS9: I hate to be with people who are unpredictable.		0.018		0.017		0.002		0.006
PNS10: I find that a consistent routine enables me to enjoy life more.	x	0.042		0.000	x	0.037		0.013
*PNS11: I enjoy the exhilaration of being in unpredictable situations.		0.008		0.016		0.002		0.017
PNS12: I become uncomfortable when the rules in a situation are not clear.	x	0.033		0.011		0.011		0.026
**Protestant Ethic (11 items)**								

PE1: Most people spend too much time in unprofitable amusements.	x	0.082		0.009		0.014		0.005
PE2: Our society would have fewer problems if people had less leisure time.	x	0.079	x	0.058		0.017		0.004
PE3: Money acquired easily is usually spent unwisely.		0.010		0.013		0.003		0.014
PE4: Most people who don't succeed in life are just plain lazy.	x	0.068	x	0.062		0.017		0.012
PE5: Anyone who is willing and able to work hard has a good chance of succeeding.	x	0.089	x	0.104	x	0.062	x	0.068
PE6: People who fail at a job have usually not tried hard enough.		0.023	x	0.051		0.018		0.023
PE7: Life would have very little meaning if we never had to suffer.		0.031		0.002		0.015		0.014
PE8: The person who can approach an unpleasant task with enthusiasm is the person who gets ahead.		0.016		0.021		0.016		0.007
PE9: If people work hard enough they are likely to make a good life for themselves.	x	0.054	x	0.081	x	0.054	x	0.049
PE10: I feel uneasy when there is little work for me to do.		0.011		0.016		0.012		0.023
PE11: A distaste for hard work usually reflects a weakness of character.	x	0.048		0.004	x	0.046		0.007
**Ring-Wing Authoritarianism (20-items)**								
RWA1: It is always better to trust the judgment of the proper authorities in government and religion, than to listen to the noisy rabble-rousers in our society who are trying to create doubt in people's minds.		0.002	x	0.042	x	0.046	x	0.042
*RWA2: People should pay less attention to the Bible and other old traditional forms of religious guidance, and instead develop their own personal standards of what is moral and immoral.	x	0.137	x	0.079	x	0.062		0.001
RWA3: What our country really needs, instead of more "civil rights," is a good stiff dose of law and order.	x	0.080	x	0.051	x	0.036	x	0.034
RWA4: Our country will be destroyed someday if we do not smash the perversions eating away at our moral fiber and traditional beliefs.	x	0.056		0.015		0.015	x	0.049
*RWA5: The sooner we get rid of the traditional family structure, where the father is the head of the family and the children are taught to obey authority automatically, the better. The old-fashioned way has a lot wrong with it.	x	0.123	x	0.048		0.002	x	0.080
*RWA6: There is nothing wrong with premarital sexual intercourse.	x	0.085		0.010		0.031	x	0.036
*RWA7: It is important to protect fully the rights of radicals and deviants.	x	0.033		0.027	x	0.038		0.000
RWA8: Obedience is the most important virtue children should learn.	x	0.039		0.011	x	0.041		0.010
*RWA9: There is no "one right way" to live your life; everybody has to create their own way.	x	0.043	x	0.073	x	0.042		0.023
*RWA10: Government, judges and the police should never be allowed to censor books.		0.004		0.021		0.025		0.020
RWA11: Some of the worst people in our country nowadays are those who do not respect our flag, our leaders, and the normal way things are supposed to be done.	x	0.093		0.016		0.005	x	0.078
RWA12: In these troubled times laws have to be enforced without mercy, especially when dealing with the agitators and revolutionaries who are stirring things up.		0.023		0.005	x	0.066		0.022

RWA13: Some young people get rebellious ideas, but as they get older they ought to become more mature and forget such things.		0.009	x	0.061		0.030	x	0.064
*RWA14: Everyone should have their own lifestyle, religious beliefs, and sexual preferences, even if it make them different from everyone else.		0.007	x	0.101		0.016	x	0.071
RWA15: The situation in our country is getting so serious, the strongest methods would be justified if they eliminated the troublemakers and got us back on our true path.	x	0.075	x	0.051		0.032		0.021
RWA16: Authorities such as parents and our national leaders generally turn out to be right about things, and the radicals and protestors are almost always wrong.	x	0.101		0.028		0.016		0.017
*RWA17: A lot of our rules regarding modesty and sexual behavior are just customs which are not necessarily any better or holier than those which other people follow.	x	0.084		0.012		0.020	x	0.056
RWA18: The real keys to the “good life” are obedience, discipline, and sticking to the straight and narrow.		0.016		0.017		0.031		0.001
RWA19: What our country really needs is a strong, determined leader who will crush evil, and take us back to our true path.		0.028		0.018		0.011	x	0.051
*RWA20: Students must be taught to challenge their parents’ views, confront the authorities, and criticize the traditions of our society.	x	0.056	x	0.056		0.019	x	0.040
**Rosenberg Self-Esteem (10-items)**								
RSE1: I feel that I am a person of worth, at least on an equal basis with others.		0.023		0.001	x	0.069	x	0.068
RSE2: I feel that I have a number of good qualities.	x	0.071		0.031	x	0.048	x	0.075
*RSE3: All in all, I am inclined to feel that I am a failure.		0.006	x	0.044		0.010		0.007
RSE4: I am able to do things as well as most other people.		0.015		0.006		0.022		0.023
*RSE5: I feel that I do not have much to be proud of.		0.012	x	0.037	x	0.036		0.021
RSE6: I take a positive attitude toward myself.	x	0.041		0.008		0.010		0.012
RSE7: On the whole, I am satisfied with myself.		0.023		0.019		0.022		0.031
*RSE8: I wish I could have more respect for myself.	x	0.061		0.004		0.014		0.026
*RSE9: I certainly feel useless at times.		0.028		0.025		0.025		0.018
*RSE10: At times I think I am no good at all.	x	0.031		0.023	x	0.037		0.027
**Self-Monitoring (18-items)**								
*SM1: I find it hard to imitate the behavior of other people.		0.007		0.009		0.018		0.023
*SM2: At parties and social gatherings, I do not attempt to do or say things that others will like.		0.009		0.007		0.013		0.022
*SM3: I can only argue for ideas which I already believe.	x	0.055	x	0.037		0.003		0.005
SM4: I can make impromptu speeches even on topics about which I have almost not information.		0.031		0.012	x	0.052		0.001
SM5: I guess I put on a show to impress or entertain others.		0.021		0.011		0.017		0.010

SM6: I would probably make a good actor.		0.020		0.015		0.021		0.004
*SM7: In a group of people I am rarely the center of attention.	x	0.044		0.021	x	0.046		0.004
SM8: In different situations and with different people, I often act like very different persons.	x	0.034		0.024		0.012		0.018
*SM9: I am not particularly good at making other people like me.		0.022		0.004		0.020	x	0.040
SM10: I'm not always the person I appear to be.	x	0.065		0.010		0.012		0.006
*SM11: I would not change my opinions (or the way I do things) in order to please someone or win their favor.		0.010		0.003	x	0.056		0.016
SM12: I have considered being an entertainer.	x	0.062		0.026		0.016		0.005
*SM13: I have never been good at games like charades or improvisational acting.		0.013		0.012		0.017		0.023
*SM14: I have trouble changing my behavior to suit different people and different situations.		0.013		0.006	x	0.043		0.021
*SM15: At a party I let others keep the jokes and stories going.		0.024		0.000		0.022		0.005
*SM16: I feel a bit awkward in company and do not show up quite as well as I should.	x	0.069		0.005	x	0.050		0.020
SM17: I can look anyone in the eye and tell a lie with a straight face (if for the right end).		0.019		0.013		0.007		0.012
SM18: I may deceive people by being friendly when I really dislike them.		0.019		0.025		0.005		0.028
**Social Dominance Orientation (12-items)**								
SDO1: Some people are just inferior to others.	x	0.046		0.006	x	0.047		0.014
SDO2: In getting what you want, it is sometimes necessary to use force against other groups.		0.002		0.016		0.010		0.016
SDO3: It's OK if some groups have more of a chance in life than others.	x	0.034		0.013	x	0.035		0.012
SDO4: To get ahead in life, it is sometimes necessary to step on others.	x	0.086	x	0.046	x	0.053		0.004
SDO5: If certain groups stayed in their place, we would have fewer problems.	x	0.038	x	0.076	x	0.032		0.010
SDO6: It's probably a good thing that certain groups are at the top and other groups are at the bottom.	x	0.050	x	0.044		0.012		0.014
SDO7: Sometimes other groups must be kept in their place.	x	0.032	x	0.036	x	0.041	x	0.047
*SDO8: All groups should be given an equal chance in life.		0.015	x	0.068		0.009		0.003
*SDO9: We should do what we can to equalize conditions for different groups.	x	0.191	x	0.037		0.001	x	0.036
*SDO10: We would have fewer problems if we treated people more equally.	x	0.059	x	0.080		0.001		0.022
*SDO11: We should strive to make incomes as equal as possible.	x	0.175	x	0.036		0.016		0.030
*SDO12: No one group should dominate society.	x	0.065	x	0.068		0.008		0.023

**Spheres of Control - Interpersonal Control (10-items)**								
*SOC-IC1: Even when I'm feeling self-confident about most things, I still seem to lack the ability to control interpersonal situations.		0.004		0.020		0.009		0.007
SOC-IC2: I have no trouble making and keeping friends.	x	0.043		0.013		0.008		0.005
*SOC-IC3: I'm not good at guiding the course of a conversation with several others.		0.016		0.026		0.013		0.013
SOC-IC4: I can usually establish a close personal relationship with someone I find sexually attractive.		0.008		0.007		0.000		0.024
SOC-IC5: When being interviewed I can usually steer the interviewer toward the topics I want to talk about and away from those I wish to avoid.		0.011		0.003	x	0.035		0.007
*SOC-IC6: If I need help in carrying out a plan of mine, it's usually difficult to get others to help.		0.011		0.017		0.008		0.009
SOC-IC7: If there's someone I want to meet I can usually arrange it.		0.014		0.012		0.005		0.002
*SOC-IC8: I often find it hard to get my point of view across to others.		0.002		0.001	x	0.063		0.021
*SOC-IC9: In attempting to smooth over a disagreement I usually make it worse.		0.001	x	0.032		0.001		0.031
SOC-IC10: I find it easy to play an important part in most group situations.		0.013		0.021		0.002		0.001
**Spheres of Control - Personal Efficacy (10-items)**								
SOC-PE1: When I get what I want it's usually because I worked hard for it.		0.006		0.005	x	0.049	x	0.053
SOC-PE2: When I make plans I am almost certain to make them work.	x	0.043	x	0.046		0.019		0.002
*SOC-PE3: I prefer games involving some luck over games requiring pure skill.		0.007		0.008	x	0.047		0.002
SOC-PE4: I can learn almost anything if I set my mind to it.		0.001		0.005		0.015		0.009
SOC-PE5: My major accomplishments are entirely due to hard work and intelligence.		0.026		0.009	x	0.066	x	0.047
*SOC-PE6: I usually don't make plans because I have a hard time following through on them.		0.013		0.024		0.021	x	0.036
SOC-PE7: Competition encourages excellence.	x	0.163		0.017		0.009		0.003
*SOC-PE8: The extent of personal achievement is often determined by chance.	x	0.130		0.006		0.009		0.022
SOC-PE9: On any sort of test or competition I like to know how well I do relative to everyone else.	x	0.045		0.002	x	0.034	x	0.032
*SOC-PE10: Despite my best efforts I have few worthwhile accomplishments.		0.015		0.002	x	0.082		0.017

Note: Table lists item codes as used in the AIID data set (https://osf.io/4dyqz/); Asterisks indicate reverse coded items. x indicates significant DIF, and r is the effect size measure.

Table S2

Uniform and Nonuniform Differential Item Functioning for ideology and extremity of items in the LISS Panel dataset, listed per scale.

Scale	ideology				extremity			
	Uniform		Nonuniform		Uniform		Nonuniform	
	sig	r	sig	r	sig	r	sig	r
Big 5 Inventory - Agreeableness (10-items)								
*cp08a021 Feel little concern for others.		0.024		0.002		0.004		0.007
cp08a026 Am interested in people.	x	0.022	x	0.019		0.015		0.005
*cp08a031 Insult people.		0.032	x	0.038		0.024	x	0.010
cp08a036 Sympathize with others' feelings.	x	0.026		0.031		0.027		0.046
*cp08a041 Am not interested in other people's problems.		0.044		0.013		0.017		0.002
cp08a046 Have a soft heart.		0.013		0.006		0.002	x	0.000
*cp08a051 Am not really interested in others.		0.016		0.019		0.009		0.039
cp08a056 Take time out for others.		0.016		0.006		0.019		0.012
cp08a061 Feel others' emotions.	x	0.010		0.021	x	0.009		0.002
cp08a066 Make people feel at ease.		0.051		0.023		0.054		0.005
**Big 5 Inventory - Openness (10-items)**								
cp08a024 Have a rich vocabulary.	x	0.027		0.008		0.014		0.021
*cp08a029 Have difficulty understanding abstract ideas.		0.035		0.004		0.016		0.017
cp08a034 Have a vivid imagination.	x	0.013	x	0.019		0.006		0.003
*cp08a039 Am not interested in abstract ideas.	x	0.066		0.032	x	0.023		0.011
cp08a044 Have excellent ideas.		0.045		0.012		0.034		0.017
*cp08a049 Do not have a good imagination.		0.009		0.011	x	0.001		0.013
cp08a054 Am quick to understand things.	x	0.001		0.004		0.035	x	0.021
cp08a059 Use difficult words.		0.027		0.021		0.016		0.030
cp08a064 Spend time reflecting on things.		0.010		0.011	x	0.008		0.024
cp08a069 Am full of ideas.		0.019		0.016		0.028		0.003

**Big 5 Inventory – Extraversion (10-items)**	x							
cp08a020 Am the life of the party.		0.047		0.006		0.015		0.019
*cp08a025 Don't talk a lot.	x	0.001		0.006		0.021		0.015
cp08a030 Feel comfortable around people.		0.040		0.011	x	0.028		0.000
*cp08a035 Keep in the background.		0.002		0.004		0.037		0.015
cp08a040 Start conversations.	x	0.022		0.010		0.006		0.010
*cp08a045 Have little to say.	x	0.043	x	0.002		0.013		0.007
cp08a050 Talk to a lot of different people at parties.	x	0.048		0.030		0.021		0.013
*cp08a055 Don't like to draw attention to myself.		0.029		0.017	x	0.018		0.027
cp08a060 Don't mind being the center of attention.		0.007		0.017		0.036		0.022
*cp08a065 Am quiet around strangers.		0.008		0.011		0.018		0.004
**Big 5 Inventory - Conscientiousness (10-items)**					x			
cp08a022 Am always prepared.		0.023		0.018		0.029		0.001
*cp08a027 Leave my belongings around.		0.030		0.016		0.004		0.007
cp08a032 Pay attention to details.	x	0.025		0.001	x	0.026		0.009
cp08a037 Make a mess of things.*	x	0.034		0.001		0.030	x	0.005
cp08a042 Get chores done right away.		0.050		0.010		0.008		0.028
*cp08a047 Often forget to put things back in their proper place.	x	0.023		0.002		0.003		0.012
cp08a052 Like order.	x	0.099	x	0.017	x	0.003	x	0.022
*cp08a057 Shirk my duties.	x	0.037		0.031		0.032		0.043
cp08a062 Follow a schedule.		0.031		0.019		0.003		0.022
cp08a067 Am exacting in my work.		0.001		0.022		0.027		0.002
**Big 5 Inventory - Neuroticism (10-items)**								
cp08a023 Get stressed out easily.		0.004		0.014		0.006		0.009
*cp08a028 Am relaxed most of the time.		0.019		0.018	x	0.001		0.015
cp08a033 Worry about things.	x	0.003	x	0.011		0.029		0.011
*cp08a038 Seldom feel blue.		0.044		0.034	x	0.000		0.019
cp08a043 Am easily disturbed.		0.006		0.010	x	0.048	x	0.017

cp08a048 Get upset easily.		0.019		0.018		0.040		0.028
cp08a053 Change my mood a lot.		0.005		0.002		0.014		0.002
cp08a058 Have frequent mood swings.	x	0.006		0.006	x	0.024		0.008
cp08a063 Get irritated easily.	x	0.053	x	0.016		0.034		0.008
cp08a068 Often feel blue.		0.052		0.043		0.004		0.015
**Need for Cognition (18-items)**								
cp08a166 I would prefer complex to simple problems	x	0.026		0.009	x	0.003		0.013
cp08a167 I like to have the responsibility of handling a situation that requires a lot of thinking	x	0.036		0.012		0.043		0.010
cp08a168 Thinking is not my idea of fun		0.071		0.006		0.000		0.009
cp08a169 I would rather do something that requires little thought than something that is sure to challenge my thinking abilities	x	0.007		0.008		0.015		0.011
cp08a170 I try to anticipate and avoid situations where there is likely chance that I will have to think in depth about something		0.031		0.005	x	0.015		0.018
cp08a171 I find satisfaction in deliberating hard and for long hours	x	0.004		0.016		0.045		0.002
cp08a172 I only think as hard as I have to	x	0.055		0.018		0.006		0.010
cp08a173 I prefer to think about small, daily projects to long-term ones		0.028		0.004	x	0.014		0.016
cp08a174 I like tasks that require little thought once I've learned them	x	0.022	x	0.013		0.030		0.010
cp08a175 The idea of relying on thought to make my way to the top appeals to me		0.095		0.046		0.003		0.016
cp08a176 I really enjoy a task that involves coming up with new solutions to problems	x	0.025		0.007		0.020		0.005
cp08a177 Learning new ways to think doesn't excite me very much		0.043		0.014		0.015	x	0.006
cp08a178 I prefer my life to be filled with puzzles that I must solve	x	0.024	x	0.007		0.006		0.037
cp08a179 The notion of thinking abstractly is appealing to me	x	0.051	x	0.055	x	0.007		0.009
cp08a180 I would prefer a task that is intellectual, difficult and important to one that is somewhat important but does not require much thought	x	0.045		0.039		0.035		0.014
cp08a181 I feel relief rather than satisfaction after completing a task that required a lot of mental effort		0.032		0.005		0.020		0.021
cp08a182 It's enough for me that somthing gets the job done; I don't care how or why it works	x	0.002	x	0.013	x	0.007		0.013
cp08a183 I usually end up deliberating issues even when they do not affect me personally		0.037		0.030		0.034		0.023

**Need to Evaluate (16-items)**								
cp08a083 I form opinions about everything		0.011		0.008	x	0.011		0.017
cp08a084 I prefer to avoid taking extreme positions	x	0.015		0.014	x	0.048		0.022
cp08a085 It's very important to me to hold strong opinions		0.059	x	0.004		0.043		0.009
cp08a086 I want to know exactly what is good and bad about everything	x	0.006		0.030		0.021	x	0.013
cp08a087 I often prefer to remain neutral about complex issues	x	0.046		0.007		0.002		0.038
cp08a088 If something does not affect me, I do not usually determine if it is good or bad	x	0.081		0.016		0.004		0.008
cp08a089 I enjoy strongly liking and disliking new things		0.045		0.003	x	0.015	x	0.000
cp08a090 There are many things for which I do not have a preference		0.026	x	0.018		0.042		0.034
cp08a091 It bothers me to remain neutral		0.011		0.031		0.009		0.013
cp08a092 I like to have strong opinions even when I am not personally involved		0.012	x	0.006		0.008		0.007
cp08a093 I have many more opinions than the average person	x	0.008		0.030	x	0.003		0.027
cp08a094 I would rather have a strong opinion than no opinion at all	x	0.073		0.027		0.041		0.014
cp08a095 I pay a lot of attention to whether things are good or bad	x	0.028		0.005		0.001	x	0.008
cp08a096 I only form strong opinions when I have to	x	0.036		0.005		0.004		0.043
cp08a097 I like to decide that new things are really good or really bad	x	0.030		0.008		0.025		0.020
cp08a098 I am pretty much indifferent to many important issues		0.054		0.013		0.019		0.018
**Positive and Negative Affect Scale – Negative affect (10-items)**							x	
cp08a147 distressed		0.017		0.016		0.004	x	0.031
cp08a149 upset		0.014		0.009	x	0.001	x	0.028
cp08a151 guilty	x	0.009	x	0.007		0.030		0.053
cp08a152 scared	x	0.033	x	0.037		0.014		0.003
cp08a153 hostile	x	0.037		0.076		0.021		0.025
cp08a156 irritable	x	0.042	x	0.019		0.002		0.009
cp08a158 ashamed		0.043		0.047		0.004		0.018
cp08a160 nervous		0.023		0.023		0.024		0.010
cp08a163 jittery		0.021		0.015		0.007	x	0.009

cp08a165 afraid		0.012		0.015		0.009		0.036
**Positive and Negative Affect Scale – Positive affect (10-items)**								
cp08a146 interested	x	0.015		0.013		0.011		0.009
cp08a148 excited		0.038		0.007	x	0.008		0.005
cp08a150 strong		0.004		0.009		0.034		0.001
cp08a154 enthusiastic	x	0.017		0.006		0.009		0.013
cp08a155 proud		0.030		0.016		0.024		0.002
cp08a157 alert	x	0.023		0.018		0.020		0.012
cp08a159 inspired		0.041		0.000		0.000		0.004
cp08a161 determined		0.013		0.025		0.010		0.002
cp08a162 attentive		0.003		0.021		0.003		0.016
cp08a164 active		0.004		0.022		0.007		0.016
**Rosenberg Self-Esteem (10-items)**	x		x					
cp08a070 I feel that I'm a person of worth, at least on an equal plane with others	x	0.052	x	0.034	x	0.004	x	0.009
cp08a071 I feel that I have a number of good qualities	x	0.065		0.037		0.060		0.029
cp08a072 All in all, I am inclined to feel that I am a failure	x	0.036		0.024	x	0.000		0.018
cp08a073 I am able to do things as well as most other people		0.029		0.006		0.055		0.001
cp08a074 I feel I do not have much to be proud of	x	0.011		0.019		0.002	x	0.011
cp08a075 I take a positive attitude towards myself		0.028		0.005		0.004		0.028
cp08a076 On the whole, I am satisfied with myself		0.021		0.027		0.005		0.016
cp08a077 I wish I could have more respect for myself		0.004		0.015		0.014		0.012
cp08a078 I certainly feel useless at times		0.020		0.017		0.025		0.014
cp08a079 At times, I think I am no good at all		0.022		0.008		0.014		0.002
**Satisfaction with Life (5-items)**	x		x					
cp08a014 In most ways my life is close to my ideal		0.044		0.044		0.026		0.002
cp08a015 The conditions of my life are excellent	x	0.011		0.003		0.002		0.017
cp08a016 I am satisfied with my life		0.034		0.010		0.004		0.017
cp08a017 So far I have gotten the important things I want in life		0.020		0.006	x	0.010		0.006

cp08a018 If I could live my life over, I would change almost nothing	0.026	0.010	0.046	0.005

Note: Table lists item codes as used in the LISS Panel data set (https://www.dataarchive.lissdata.nl/study_units/view/15); Asterisks indicate reverse coded items. x indicates significant DIF, and r is the effect size measure.

Table S4

Summary of ideological difference tests using the raw and corrected versions of the scales in the Attitudes 2.0 dataset (USA). Rows are sorted by the percentage of items with DIF due to political ideology

Measure	Unadjusted Scale Ideology			Adjusted Scale Ideology			Absolute Value of Size of Difference	Conclusions Changed?
	B	SE	P	B	SE	P		
Social Dominance Orientation	2.194	0.151	**	1.894	0.145	**	0.300	no
Humanitarianism-Egalitarianism	-2.050	0.158	**	-3.004	0.218	**	0.954	no
Big 5 Inventory - Openness	-0.621	0.093	**	-0.240	0.093	**	0.381	no
Ring-Wing Authoritarianism	4.156	0.181	**	3.605	0.169	**	0.551	no
Balanced Inventory of Desirable Responding - Impression Management	0.470	0.087	**	0.543	0.097	**	0.073	no
Protestant Ethic	1.930	0.121	**	1.755	0.119	**	0.175	no
Bayesian Racism	2.167	0.134	**	1.933	0.120	**	0.234	no
Need for Cognitive Closure - Closed-mindedness	1.038	0.125	**	0.837	0.125	**	0.201	no
Rosenberg Self-Esteem	0.499	0.069	**	0.528	0.069	**	0.029	no
Need for Cognitive Closure - Ambiguity	0.514	0.093	**	0.415	0.114	**	0.099	no
Balanced Inventory of Desirable Responding - Self Deception	0.747	0.101	**	0.632	0.094	**	0.115	no
Belief in a Just World	1.310	0.108	**	0.946	0.109	**	0.364	no
Big 5 Inventory - Extraversion	0.089	0.088	ns	0.101	0.082	ns	0.012	no
Need for Cognition	-0.604	0.093	**	-0.265	0.073	**	0.339	no
Need for Cognitive Closure - Order	0.772	0.091	**	0.753	0.085	**	0.019	no
Personal Need for Structure	0.642	0.089	**	0.496	0.084	**	0.146	no
Spheres of Control - Personal Efficacy	0.688	0.092	**	0.471	0.089	**	0.217	no
Big 5 Inventory - Neuroticism	-0.408	0.094	**	-0.323	0.084	**	0.085	no
Self-Monitoring	-0.146	0.097	ns	-0.092	0.088	ns	0.054	no
Need for Cognitive Closure - Predictability	0.646	0.095	**	0.542	0.087	**	0.104	no

Big 5 Inventory - Conscientiousness	0.399	0.074	**	0.389	0.071	**	0.010	no
Spheres of Control - Interpersonal Control	0.138	0.086	ns	0.111	0.084	ns	0.027	no
Need for Cognitive Closure - Decisiveness	0.529	0.088	**	0.524	0.080	**	0.005	no
Big 5 Inventory - Agreeableness	0.050	0.100	ns	0.042	0.093	ns	0.008	no

Note: Conclusions changed is marked as “yes” when there are differences in significance or direction and significance between the unadjusted and the adjusted scale. For the final model for Humanitarianism-Egalitarianism, all items show some DIF. For model identification and convergence, we removed DIF effects of item 6 and 9 (the smallest DIF in comparison to all other items).

Table S5

Summary of ideological difference tests using the raw and corrected versions of the scales in the LISS Panel (NL). Rows are sorted by the percentage of items with DIF due to political ideology

Measure	Unadjusted Scale Ideology			Adjusted Scale Ideology			Absolute Value of Size of Difference	Conclusions Changed?
	B	SE	P	B	SE	P		
Need to Evaluate	0.422	0.090	**	0.305	0.082	**	0.117	no
Need for Cognition	-0.348	0.087	**	-0.307	0.081	**	0.041	no
Big 5 Inventory - Extraversion	0.308	0.086	**	0.25	0.080	**	0.058	no
Big 5 Inventory - Conscientiousness	0.571	0.088	**	0.388	0.083	**	0.183	no
Rosenberg Self-Esteem	0.032	0.077	ns	0.095	0.078	ns	0.063	no
Big 5 Inventory - Agreeableness	-0.564	0.090	**	-0.604	0.084	**	0.040	no
Big 5 Inventory - Openness	-0.392	0.089	**	-0.379	0.083	**	0.013	no
Positive and Negative Affect Scale – Negative affect	0.165	0.079	*	0.114	0.076	ns	0.051	yes
Satisfaction with Life	0.406	0.074	**	0.453	0.076	**	0.047	no
Big 5 Inventory - Neuroticism	-0.168	0.078	*	-0.135	0.08	ns	0.033	yes
Positive and Negative Affect Scale – Positive affect	0.327	0.085	**	0.341	0.081	**	0.014	no

Note: Conclusions changed is marked as “yes” when there are differences in significance or direction and significance between the unadjusted and the adjusted scale.

Table S6

Summary of ideological extremity different tests using the raw and corrected versions of the scales in the Attitudes 2.0 dataset (USA). Rows are sorted by the percentage of items with DIF due to political ideology

Measure	Unadjusted Scale Ideological extremity			Adjusted Scale Ideological extremity			Absolute Value of Size of Difference	Conclusions Changed?
	B	SE	P	B	SE	P		
Social Dominance Orientation	-0.248	0.074	**	-0.223	0.076	**	0.025	no
Humanitarianism-Egalitarianism	0.540	0.085	**	0.441	0.088	**	0.099	no
Big 5 Inventory - Openness	0.340	0.062	**	0.379	0.064	**	0.381	no
Ring-Wing Authoritarianism	-0.144	0.056	**	-0.236	0.063	**	0.092	no
Balanced Inventory of Desirable Responding - Impression Management	0.045	0.057	ns	0.043	0.063	ns	0.002	no
Protestant Ethic	-0.241	0.063	**	-0.009	0.068	ns	0.232	yes
Bayesian Racism	-0.074	0.068	ns	-0.119	0.070	ns	0.045	no
Need for Cognitive Closure - Closed-mindedness	0.274	0.076	**	0.155	0.079	ns	0.119	yes
Rosenberg Self-Esteem	0.127	0.047	**	0.089	0.047	ns	0.038	yes
Need for Cognitive Closure - Ambiguity	-0.015	0.060	ns	-0.166	0.083	*	0.151	yes
Balanced Inventory of Desirable Responding - Self Deception	0.057	0.063	ns	-0.016	0.066	ns	0.073	no
Belief in a Just World	-0.327	0.065	**	-0.088	0.110	ns	0.239	yes
Big 5 Inventory - Extraversion	-0.015	0.059	ns	0.008	0.056	ns	0.012	no
Need for Cognition	0.462	0.062	**	-0.007	0.049	ns	0.469	yes
Need for Cognitive Closure - Order	-0.226	0.058	**	-0.208	0.057	**	0.018	no
Spheres of Control - Personal Efficacy	-0.056	0.058	ns	0.009	0.067	ns	0.065	no
Big 5 Inventory - Neuroticism	-0.030	0.061	ns	-0.005	0.057	ns	0.025	no
Self-Monitoring	0.038	0.064	ns	0.066	0.059	ns	0.028	no
Need for Cognitive Closure - Predictability	-0.029	0.061	ns	-0.045	0.058	ns	0.016	no

Big 5 Inventory - Conscientiousness	-0.044	0.05	ns	-0.045	0.049	ns	0.001	no
Personal Need for Structure	-0.085	0.058	ns	-0.099	0.056	ns	0.014	no
Spheres of Control - Interpersonal Control	0.225	0.058	**	0.195	0.057	**	0.030	no
Need for Cognitive Closure - Decisiveness	0.128	0.058	*	0.057	0.055	ns	0.071	yes
Big 5 Inventory - Agreeableness	-0.076	0.065	ns	-0.046	0.062	ns	0.030	no

Note: Conclusions changed is marked as "yes" when there are differences in significance or direction and significance between the unadjusted and the adjusted scale. For the final model for Humanitarianism-Egalitarianism, all items show some DIF. For model identification and convergence, we removed DIF effects of item 6 and 9 (the smallest DIF in comparison to all other items).

Table S7

Summary of ideological different tests using the raw and corrected versions of the scales in the LISS Panel. Rows are sorted by the percentage of items with DIF due to political ideology

Measure	Unadjusted Scale Ideological extremity B	SE		Adjusted Scale Ideological extremity B	SE		Absolute Value of Size of Difference	Conclusions Changed?
Need to Evaluate	0.815	0.084	**	0.684	0.072	**	0.131	no
Need for Cognition	0.393	0.079	**	0.244	0.069	**	0.149	no
Big 5 Inventory - Extraversion	0.256	0.074	**	0.253	0.068	**	0.003	no
Big 5 Inventory - Conscientiousness	-0.025	0.073	ns	-0.038	0.069	ns	0.013	no
Rosenberg Self-Esteem	0.191	0.066	**	0.148	0.066	*	0.043	no
Big 5 Inventory - Agreeableness	0.066	0.077	ns	0.016	0.069	ns	0.050	no
Big 5 Inventory - Openness	0.467	0.079	**	0.399	0.07	**	0.068	no
Positive and Negative Affect Scale – Negative affect	-0.014	0.067	ns	-0.017	0.064	ns	0.003	no
Satisfaction with Life	-0.056	0.063	ns	-0.037	0.065	ns	0.019	no
Big 5 Inventory - Neuroticism	-0.035	0.067	ns	0.023	0.07	ns	0.033	no
Positive and Negative Affect Scale – Positive affect	0.203	0.072	**	0.203	0.069	**	0	no

Note: Conclusions changed is marked as “yes” when there are differences in significance or direction and significance between the unadjusted and the adjusted scale.
